# Incidence of acute myocardial infarction in people with diabetes compared to those without diabetes: a systematic review

**DOI:** 10.1186/s13643-026-03089-x

**Published:** 2026-02-09

**Authors:** Maria Narres, Tatjana Kvitkina, Heiner Claessen, Ellen Ubach, Georg Wolff, Maria-Inti Metzendorf, Bernd Richter, Michael Roden, Andrea Icks

**Affiliations:** 1 Institute for Health Services Research and Health Economics, German Diabetes Center, Leibniz Center for Diabetes Research at Heinrich-Heine-University Düsseldorf, Auf’M Hennekamp 65, Düsseldorf, 40225 Germany; 2 Institute for Health Services Research and Health Economics, Centre for Health and Society, Medical Faculty and University Hospital Düsseldorf, Heinrich Heine University Düsseldorf, Düsseldorf, Germany; 3 German Center for Diabetes Research, Partner Düsseldorf, München-Neuherberg, Germany; 4 Clinic of Cardiology, Pulmonology, and Vascular Medicine, Medical Faculty and University Hospital Düsseldorf, Heinrich Heine University Düsseldorf, Düsseldorf, Germany; 5 Clinic of Cardiology, Klinikum Ibbenbüren, Stiftung Mathias-Spital Rheine, Ibbenbüren, Germany; 6 Institute of General Practice, Medical Faculty of the Heinrich-Heine-University Düsseldorf, Düsseldorf, Germany; 7 Department of Endocrinology and Diabetology, Medical Faculty and University Hospital Düsseldorf, Heinrich-Heine-University Düsseldorf, Düsseldorf, Germany; 8 Institute for Clinical Diabetology, German Diabetes Center, Leibniz Center for Diabetes Research at Heinrich Heine University, Düsseldorf, Germany

**Keywords:** Diabetes mellitus, Cardiovascular disease, Myocardial infarction, Incidence, Time trend, Epidemiology, Systematic review

## Abstract

**Background:**

Although diabetes mellitus is an established risk factor for acute myocardial infarction (AMI), epidemiological studies showed wide variations in the incidence of AMI in people with diabetes and inconsistent time trends. The objectives of the present systematic review were as follows: (i) to analyze the age–sex-adjusted incidence of both non-fatal and fatal AMI in people with diabetes compared to those without diabetes, (ii) to investigate corresponding time trends, and (iii) to identify sex differences.

**Methods:**

A systematic literature search was performed in the literature databases MEDLINE, Embase, and LILACS until July 19, 2023, to identify population-based studies reporting the incidence of AMI in people with diabetes compared to those without diabetes according to our predefined inclusion criteria.

**Results:**

In total, 28 population-based cohort studies were included in this review. In women with diabetes, the incidence of AMI ranged from 102 to 690 per 100,000 person-years, and in men with diabetes from 206 to 1630. Estimates comparing people with and without diabetes ranged from 1.55 (95% CI 1.44–1.67) to 14.37 (8.43–24.47) in women and from 1.33 (1.18–1.51) to 4.17 (2.72–6.37) in men. Over the past four decades, the incidence of AMI declined in almost all studies in people without diabetes, but only in half of the studies in people with diabetes. There was considerable heterogeneity with regard to the definition of AMI, the population with diabetes, and geographic differences.

**Conclusion:**

Incidence of AMI in people with diabetes remained significantly higher than in those without diabetes. A reduction in the incidence of AMI over time was observed in some, but not all reviewed studies in people with diabetes. There was no discernible trend of estimates comparing people with and without diabetes. These findings underscore the necessity for additional initiatives to prevent coronary heart disease in people with diabetes. The observed discrepancy in study results is presumably attributable to variations in the population and regional contexts, as well as to disparate methodological approaches. More standardized studies employing comparable methodologies are therefore required.

**Systematic review registration:**

PROSPERO CRD42 02014 5562

**Supplementary Information:**

The online version contains supplementary material available at 10.1186/s13643-026-03089-x.

## Introduction

Diabetes mellitus is a chronic disease with an estimated global prevalence of 10.5% among adults, or 588.7 million people worldwide [1]. There is evidence that diabetes mellitus has a strong negative impact on the cardiovascular system, increasing the risk of acute myocardial infarction (AMI) compared to people without diabetes [1–5]. Moreover, mortality after AMI among people with diabetes was twice as high as in people without [6, 7]. The incidence of AMI in people with diabetes therefore remains an important indicator of diabetes care and a pertinent public health problem [8]. The 1989 St. Vincent declaration aimed to reduce morbidity and mortality from coronary heart diseases (CHD) among people with diabetes [9]. A number of studies have reported a decreasing time trend in the incidence of AMI among people with diabetes in recent decades [10]. However, a recent study conducted in the USA found an increased risk of hospitalization for AMI in young and middle-aged people following a decrease in the 1990 s and 2010s. In contrast, this risk remained stable for people 65 years and older [11]. Furthermore, it remains unclear whether the efforts to prevent cardiovascular disease (CVD) and changing cardiovascular management in recent decades have affected the time trends of AMI in people with and without diabetes accordingly. In addition, several published studies have shown marked variations in the incidence of AMI in people with and without diabetes [10, 12–16]. However, these studies were heterogeneous regarding study population, definition of AMI, definition of population with diabetes, and statistical methods. Thus, it remains unclear whether these variations are related to differences in diabetes care between countries, or are perhaps partly due to methodological discrepancies between studies. Several reviews have been published on CVD risk in people with diabetes [3, 17–19]. Nevertheless, some of them primarily analyzed sex differences of CHD [18] or mortality after AMI [7], whilst others focused only on certain regions [20] or were not population-based [21, 22]. The findings from a recent systematic review on this topic demonstrated a 1.7 higher risk for AMI in people with type 2 diabetes compared to controls [1]. However, the studies reviewed used different definitions of AMI and some were not population-based, while others analyzed only non-fatal AMI [1]. Given the increasing prevalence of diabetes worldwide, the substantial number of individuals affected by both diabetes and cardiovascular disease (CVD), and the controversial results of previous studies, it is highly relevant to ascertain the incidence both of non-fatal and fatal AMI in people with diabetes compared to those without diabetes and to examine the time trends (temporal changes of incidence) from population-based studies.

To our knowledge, no systematic review of this research question has been performed. To address this research gap, we conducted the present systematic review with the following objectives: (i) to analyze the incidence of AMI in people with diabetes compared to those without diabetes, (ii) to investigate corresponding time trends, and (iii) to identify potential sex differences in the incidence of AMI.

## Methods

We conducted a systematic review in accordance with the standards recommended by the Meta-analysis Of Observational Studies in Epidemiology (MOOSE) guidelines [23] and reported it according to the Preferred Reporting Items for Systematic Reviews and Meta-Analyses (PRISMA) statements [24]. The PRISMA 2020 checklist is provided in the supplemental information (Table S1). The protocol of the current systematic review was registered in PROSPERO (CRD42 02014 5562) and was published previously [25].

### Data sources and searches

A systematic literature search was performed in the MEDLINE, Embase, and LILACS literature databases until 19 July 2023. This database selection adheres to recommendations on searching for epidemiological studies [26]. Moreover, we additionally searched reference lists of review articles and relevant studies to identify potentially eligible studies. A comprehensive systematic search strategy was designed by an experienced information scientist (M-IM) and tested against 20 known relevant references from previous systematic reviews. All search strategies are presented in the supplemental information (File S2). All database records yielded by the search were exported to EndNote and duplicates were removed.

### Eligibility criteria

#### Inclusion criteria

##### Types of studies

We included population-based cohort studies that compared the incidence of AMI between people with and without diabetes using either prospective or retrospective designs.

##### Study population

The study population had to be defined using official statistics, for instance, citizens of a country or a defined administrative region or all those insured by a statutory health insurance provider.

The population with diabetes had to be precisely described (register, survey data, estimation based on age-sex-specific prevalence data). Study populations could include people with (a) type 1 diabetes or (b) type 2 diabetes or (c) without distinguishing between diabetes types. Tables 1, 2, and 3 provide information regarding diabetes type for each study. The population without diabetes was only taken into account when comparing the incidence rates of people with diabetes.

**Table 1:** Study characteristics

Study, year, country	Study period/study design	Entire study population, sample size, age	Type of DM	Definition of DM in population at risk	Definition of AMI	Data source for AMI	Exclusion criteria in population at risk	Time trend
**I. Incidence rate**								
Ballotari, P 2017 Italy [27]	2012–2014 prospective	Inhabitants from the Reggio Emilia region in Italy (total approx. 0.5 million inhabitants), n = 356,191 Age 30–84 years Median age at baseline Women DM 70 (61–77) Women no DM 50 (40–64) Men DM 67 (58–74) Men no DM (48 (39–61)	Prevalent type 2 DM (known & unknown)	Reggio Emilia Diabetes Registry: co-payment exemption due to DM; hospitalization with DM diagnosis; one HbA1c test ≥ 6.5% (48 mmol/mol); prescription for glucose-lowering drugs; outpatient diagnosis by diabetologist; cause of death by ICD-10 codes E10-E14 n = 24,348	Non-fatal AMI (ICD-9 codes 410–411) or death from AMI (ICD-10 codes I21, I22, I24.8, I24.9)	Reggio Emilia hospital discharge database and death register	People with CHD at baseline were not excluded	N/A
Barengo, N 2008 Finland [12]	Cohort 1: 1972–1977 + 10 years follow-up Cohort 2: 1982–1987 + 10 years follow-up prospective	Random sample (6.6%) of population of North Karelia and Kuopio provinces in east Finland n = 35,014 Age 25–64 years Mean age N/A	Incident and prevalent non-differentiated DM (known)	Incident DM: new cases of DM during follow-up Prevalent DM: DM at baseline National drug reimbursement records of the Social Insurance Institution and the hospital admission records of the National Hospital Discharge Register by computer-based record linkage DM prevalence: cohort 1 women 3% men 3% cohort 2 women 6% men 5% n = N/A	First non-fatal AMI (ICD-9 410–411) or fatal CHD event (ICD-9 410–414)	National hospital discharge register, mortality register of the Finnish Statistical Office	People with CHD at baseline were not excluded	IR + RR +
Carson, A 2014 USA [13]	Two prospective cohort studies: ARIC study 1987–1996, REGARD study 2003–2009	Only White and Black participants from ARIC and REGARD studies; ARIC: participants from four communities—Forsyth County (North Carolina), Jackson (Mississippi), suburbs of Minneapolis (Minnesota), Washington County (Maryland) n = 14,825 REGARD: participants from 48 U.S. states and the District of Columbia n = 12,712 Age 45–64 years Mean age at baseline ARIC study DM 55.5 (5.7) No DM 53.8 (5.7) REGARD study DM 57.9 (4.6) No DM 56.9 (5.0)	Prevalent non-differentiated DM (known & unknown)	Self-reported physician-diagnosed DM with concomitant use of insulin or oral glucose-lowering drugs, fasting glucose ≥ 7.0 mmol/L (126 mg/dL), or occasional glucose ≥ 11.1 mmol/L (200 mg/dL), ARIC n = 1,673 REGARD n = 2,297	Definite, probable, or silent AMI or definite CHD death	Medical records, death certificates, autopsy reports, and National Death Index	CHD at baseline	IR + HR +
de Jong, M 2020 United Kingdom [5]	2006–2018 prospective	Participants from UK Biobank n = 471,399 Age 40–69 years Mean age at baseline Women prevalent DM 58.3 (7.7) incident DM 58.9 (6.9) pre-DM 59.8 (6.6) no-DM 55.6 (8.0) Men prevalent DM 59.4 (7.2) incident DM 57.3 (7.8) pre-DM 58.6 (7.6) no-DM 55.7 (8.3)	Incident and prevalent non-differentiated DM (known & unknown)	Incident DM: no previous DM diagnosis, HbA1c 6.5% (48 mmol/mol), no use of glucose-lowering drugs n = 3,054 Prevalent DM: self-reported DM and/or use of glucose-lowering drugs n = 21,656 Pre-diabetes: no previous diagnosis of DM, HbA1c 5.7% (39 mmol/mol)—6.5% (48 mmol/mol), no use of glucose-lowering drugs) n = 54,425	Non-fatal or fatal AMI defined by ICD-10 codes I21-I21.4, I21.9, I22-I22.1, I22.8, I22.9, I23-I23.6, I23.8, I24.1, and I25.2	Hospitalization data from England, Scotland, and Wales and the National Death Registry	AMI, stroke, angina pectoris at baseline	N/A
Dybjer, E 2021 Sweden [28]	1959/1961 −2018 prospective	Men with type 1 DM and control group of men without DM from the region of Scania, southern Sweden n = 589 Men born 1934–1943 Mean age N/A	Prevalent type 1 DM	Men with type 1 DM diagnosed before 18 years from hospital registers in the region of Scania n = 120	Non-fatal or fatal AMI defined by ICD-10 I21, ICD-9 and ICD-8 410, ICD-7 420	Swedish National Patient Register, Swedish Cause of Death Registry	CHD at baseline	N/A
Folsom, A 1997 USA [29]	1987–1995 prospective	Participants (26% Black) from ARIC study: four communities—Forsyth County (North Carolina), Jackson (Mississippi), suburbs of Minneapolis (Minnesota), Washington County (Maryland) n = 13,446 Age 45–64 years Mean age N/A	Prevalent non-differentiated DM (known & unknown)	Self-reported physician-diagnosed DM or fasting glucose ≥ 7.0 mmol/L (126 mg/dL), or occasional glucose ≥ 11.1 mmol/L (200 mg/dL) or use of glucose-lowering drugs DM prevalence: Black women 18% Other women 6% Black men 15% Other men 7% n = N/A	Definite, probable, or silent AMI or definite CHD death	Hospital discharge reports, death certificates, autopsy reports	CHD at baseline	N/A
Fujishima, M 1996 Japan [30]	1988–1993 prospective	Residents from Hisayama city in southern Japan n = 2,427 Age 40–79 years Mean age N/A	Prevalent NIDDM/ type 2 DM (known & unknown)	DM: Fasting glucose ≥ 7.8 mmol/L or OGTT ≥ 11.1 mmol/L Total n = 260 Women n = 123 Men n = 137 IGT: Fasting glucose < 7.8 mmol/L and OGTT 7.8–11 mmol/L n = 474	Acute or silent AMI and sudden cardiac death	Autopsy reports, clinical diagnoses	AMI and stroke at baseline	N/A
Hyvarinen, M 2009 Finland [31]	Finland 1987–2006 Sweden 1986–2004 retrospective	Finnish and Swedish cohorts from Decode study Total n = 9,278 Women n = 5,111 Men n = 4,167 Age 40–69 years Median age at baseline Women 55.1 Men 55.1	Prevalent non-differentiated DM (known & unknown)	DM: Diagnosis DM at baseline or fasting glucose ≥ 7.0 mmol/L and/or OGTT ≥ 11.1 mmol/L Total n = 826 Women n = 384 Men n = 442	Non-fatal MI (410–411, I21 to I22) and CHD deaths (410–414, I20-I25)	National Registry of Hospital Discharges, National Registry of Causes of Death	CVD at baseline	N/A
Icks, A 2009 Germany [15]	1985–2006 prospective	Participants from Augsburg and the regions of Augsburg and Aichach-Friedberg n = approx. 400,000 Age 25–74 years Mean age during study period Women DM 66.8 (6.7) No-DM 64.2 (8.8) Men DM 63.3 (8.2) No-DM 59.9 (9.9)	Prevalent non-differentiated DM (known)	Self-reported physician-diagnosed DM and use of glucose-lowering drugs DM prevalence: women 3.5%—3.7% men 3.7%—4.6% n = N/A	Definite or probable AMI; non-fatal AMI according to WHO criteria or CHD death (before admission to hospital or within 24 h of hospitalisation)	MONICA/KORA heart attack registry in Augsburg	People with CHD at baseline were not excluded	IR + RR +
Juutilainen, A 2004 Finland [32]	1982–1996 prospective	People with DM and random control sample from Turku University Hospital district in west Finland and in the Kuopio University Hospital district in east Finland n = 2,135 Age 45–64 years Mean age at baseline Women DM 58.7 (4.9) No-DM 54.8 (5.5) Men DM 56.9 (5.1) No-DM 54.4 (5.6)	Prevalent type 2 DM (known)	National drug reimbursement register Total n = 835 Women n = 406 Men n = 429	First definite or possible non-fatal AMI or CHD death	Hospital and autopsy reports and cause-of-death register	CVD (verified prior AMI, stroke, non-traumatic LEA) at baseline	N/A
Liu, F 2017 China [33]	2000–2008 prospective	Participants from the China Multicenter Collaborative Study of Cardiovascular Epidemiology study and the China Cardiovascular Health study n = 18,610 Age 35–74 years Mean age at baseline DM 52.8 (9.5) No-DM 47.6 (8.9) IFG 50.9 (9.7)	Prevalent non-differentiated DM (known & unknown)	Self-reported physician-diagnosed DM or fasting glucose ≥ 126 mg/dL or use of blood glucose-lowering drugs n = 1,004 IFG: Fasting glucose 100–125 mg/dL n = 3,607	Non-fatal AMI or CHD death	Hospital records, autopsy results, death certificates from the local health department or police department	AMI and stroke at baseline	N/A
Lundberg, V 1997 Sweden [34]	1989–1993 prospective	Random sample of inhabitants from Norrbotten and Vasterbotten counties (northern Sweden), approx. 200,000 inhabitants n = 2,432 Age 35–64 years Mean age N/A	Prevalent non-differentiated DM (known & unknown)	Based on two surveys: self-reported DM, in random sample OGTT: fasting glucose ≥ 7.7 mmol/L, 2 h ≥ 11.1 mmol/L DM prevalence: Total 4.7% Women 4.4% Men 5.0% n = N/A	AMI according to MONICA criteria Non-fatal AMI: only definite infarcts fatal AMI: definite, possible and unclassifiable AMI death within 28 days	Hospital discharge reports, primary care physician reports, death certificates with ICD codes 410–414	AMI at baseline	N/A
Matuleviciene, V 2017 Sweden [35]	1998–2011 prospective	Participants from the Swedish Diabetes Registry and matched controls from the population registry n = 197,868 Age ≥ 18 years Mean age at baseline DM 35.3 (14.3) Controls 35.1 (14.2)	Prevalent type 1 DM	Swedish Diabetes Registry: treatment with insulin and DM diagnosis at age ≤ 30 years n = 33,170	Non-fatal AMI (ICD10 I21, ICD9 410) or CHD death (ICD10 I20-I25, ICD9 410–414)	Swedish registries for inpatient stays and causes of death	AMI at baseline	IR- HR +
Millett, E 2018 United Kingdom [36]	2006–2016 prospective	Participants from UK Biobank n = 471,998 Age 40–69 years Mean age at baseline Women all 56.2 (8.0) Men all 56.3 (8.2)	Prevalent type 1 DM and prevalent type 2 DM (known)	Known prevalent type 1 DM: Age at DM diagnosis < 30 years and treatment with insulin n = 1,031 Known prevalent type 2 DM: Age at DM diagnosis ≥ 30 years and no insulin treatment n = 20,483	First known AMI (fatal or non-fatal) ICD codes I21, I22, I23, I24.1, I25.2	Office for National Statistics hospitalization and death registry data	AMI, stroke, angina pectoris at baseline	N/A
Moe, B 2015 Norwegen [37]	1995–2008 prospective	Inhabitants from the Nord-Trøndelag region Total n = 55,534 Women n = 29,305 Men n = 26,229 Age ≥ 20 years Mean age at baseline Women DM 63.4 (14.9) No-DM 46.8 (16.1) Men DM 60.4 (14.6) No-DM 47.0 (15.7)	Prevalent non-differentiated DM (known & unknown)	Self-reported DM or non-fasting glucose ≥ 11 mmol/L n = 1,366	Non-fatal and fatal AMI (ICD-9 410 and ICD-10 I21)	Medical records, national registry of causes of death	AMI at baseline	N/A
Oğuz, A 2023 Türkiye [38]	2008–2020 prospective	Randomized sampling Participants from selected households from eight regions in Türkiye: Kocaeli, Aydın, Nevşehir, Antalya, Samsun, Malatya, Gaziantep, Istanbul n = 3,816 Age 35–70 years Mean age at baseline DM 54.8 (8.4) No-DM 49.3 (9.0)	Prevalent non-differentiated DM (known & unknown)	Fasting plasma glucose level of ≥ 7.0 mmol/L (126 mg/dL), self-reported physician-diagnosed DM and use of glucose-lowering drugs n = 485	Non-fatal and fatal AMI ICD-10 I21	Prospective Urban and Rural Epidemiological Türkiye study	CHD at baseline	N/A
Rautio, A 2005 Sweden [39]	1989–2000 prospective	Inhabitants from two northernmost counties of Sweden (approx.200,000) n = approx. 200,000 Age 35–64 years Mean age N/A	Prevalent non-differentiated DM (known & unknown)	DM based on four surveys: self-reported DM, in random sample OGTT: fasting glucose ≥ 7.7 mmol/L, 2 h ≥ 11.1 mmol/L DM prevalence: Women 2.4% Men 3.6%	Non-fatal AMI and fatal AMI according MONICA criteria	The Northern Sweden MONICA Project registry	AMI at baseline	IR + RR +
Read, SH 2019 United Kingdom [4]	2006–2015 retrospective	Nationwide data from Scotland n = N/A Age 30–89 years Mean age N/A	Prevalent type 2 DM (known)	Scottish Care Information—Diabetes register: daily links from both primary care and secondary care systems n = N/A	Non-fatal AMI: hospital admissions (I21-I22) Fatal AMI: CHD death (I20-I25.4)	Non-fatal AMI: Scottish Morbidity Records database that records all hospital admissions in Scotland Fatal AMI: national death records	CHD at baseline	IR + RR +
Saito, I 2011 Japan [40]	1990–2006 prospective	11 areas with public health-center areas in Japan n = 31,192 Age 40–69 years Mean age at baseline DM 56.6 Borderline 54.4 No DM 53.7	Prevalent non-differentiated DM (known & unknown)	DM: Fasting glucose ≥ 7.0 mmol/L, non-fasting glucose ≥ 11.1 mmol/L or glucose-lowering drugs n = 1,256 Borderline: Fasting glucose 5.6–6.9 mmol/L and non-fasting glucose of 7.8–11.0 mmol/L n = 4,744	Non-fatal AMI: definite or possible AMI according to Monica criteria, CHD death (ICD-10 I21-I23, I46, I50)	Hospital records and death certificates	AMI, stroke, angina pectoris, cancer at baseline	N/A
Tancredi, M 2019 Sweden [41]	1998–2012 prospective	Participants from the Swedish National Diabetes Registry and random controls from the Swedish general population n = 2,605,199 Mean age at baseline DM 65.0 (12.7) Controls 64.7 (12.6)	Prevalent type 2 DM (known)	Swedish National Diabetes Registry: treatment with diet with or without oral antihyperglycemic drugs or insulin (the latter only in patients ≥ 40 years of age at DM diagnosis) n = 431,579	Non-fatal AMI (ICD-9 410, and ICD-10 I21) or CHD death (ICD-9 410–414, and ICD-10 I20-I25)	Swedish Patient Registry and Registry of Causes of Death	AMI at baseline	IR- HR +
Vimalananda, V 2014 USA [42]	1989–2002 prospective	White and Black participants, random sample of Medicare-eligible residents from four US counties (Sacramento County, Washington County, Forsyth County, Pittsburgh) Total n = 4,817 White women n = 2,447 White men n = 1,625 Black women n = 476 Black men n = 279 Age ≥ 65 years Mean age at baseline White women DM 72.5 (5.9) controls 72.2 (5.3) White men DM 72.7 (5.7) controls 72.6 (5.5) Black women DM 72.9 (5.3) controls 73.1 (5.8) Black men DM 72.2 (4.9) controls 72.5 (5.6)	Prevalent non-differentiated DM (known & unknown)	Treatment with insulin or oral antidiabetic drugs, fasting ≥ 7 mmol/L or non-fasting occasional glucose ≥ 11.1 mmol/L Total n = 681 White women n = 251 White men n = 105 Black women n = 111 Black men n = 72	Non-fatal AMI or CHD death	Medical records, death certificates	CHD, heart failure, atrial fibrillation at baseline	N/A
Wannamethee, S 2011 United Kingdom [43]	1998–2008 prospective	White Europeans (> 99%) from 24 UK cities, from the British Regional Heart Study, only men n = 3,611 Age 60–79 years Mean age at baseline Onset DM < 60 years 66.8 Onset DM ≥ 60 years 69.7 Controls 68.5	Prevalent non-differentiated DM (known & unknown)	Self-reported confirmed physician diagnosis of DM or fasting glucose ≥ 126.1 mg/dL early-onset DM (diagnosed before 60 years) late-onset diabetes (diagnosed at ≥ 60 years or undiagnosed DM) n = 414	Non-fatal AMI according WHO criteria or CHD death	Non-fatal AMI: general practitioner reports, review of patient notes, postal questionnaires Fatal AMI: National Health Service registry	AMI at baseline	N/A
Wright, A 2019 United Kingdom [44]	2006–2015 retrospective	Participants from the Clinical Practice Research Datalink People with T2DM and matched controls n = 340,894 Mean age at baseline Women DM 63.9 (14.3) Controls 63.7 (14.4) Men DM 61.3 (13.0) Controls 60.8 (13.0)	Incident type 2 DM (known)	Read codes in the electronic health record n = 63,718	Fatal or non-fatal MI	Hospitalization data (Hospital Episode Statistics), Fatal AMI: national mortality data (Office for National Statistics)	CHD at baseline	IR- HR +
**II. Cumulative incidence**								
Haffner, S 1998 Finland [45]	1982–1989 prospective 7Y CumI	Patients with DM from the Finnish Social Security + random control sample from the population register of Kuopio or Turku n = 2,194 Age 45–64 years Mean age at baseline DM 57.9 (0.2) No-DM 54.6 (0.6)	Prevalent type 2 DM (known)	Central drug reimbursement registry n = 890	Non-fatal and fatal AMI; definite or possible AMI according WHO Criteria	Hospital records and autopsy reports, hospital discharge registers	AMI at baseline	N/A
Nørgaard, C 2022 Denmark [46]	1997–2014 retrospective 5Y CumI	Nationwide data from Denmark: people with newly diagnosed diabetes and matched controls from the general population n = 2,237,928 Age 30–89 years Median age at baseline (IQR) 1997–2002 DM 60.2 (51.5, 70.4) Controls 61.6 (52.3,71.8) 2003–2008 DM 60.5 (51.5, 69.2) Controls 61.0 (51.9,70.0) 2009–2014 DM 61.1 (51.5, 69.2) Controls 61.3 (51.7,69.4)	Incident type 2 DM (known)	First ever prescription for a glucose-lowering drug from The National Prescription Registry (Danish Registry of Medicinal Product Statistics) or diagnosis of type 2 DM during a hospital contact based on The Danish National Patient Registry n = 203,448	Non-fatal and fatal AMI ICD-8: 410 ICD-10 I21-I22	The Danish National Patient Registry for discharge diagnoses and Danish Causes of Death Registry	CVD (IHD, PAD, ischemic stroke, heart failure) at baseline	CumI + HR +
Rosengren, A 1989 Sweden [47]	1974–1983 prospective 7Y CumI	Men from Gothenburg born between 1915 and 1925, excluding 1923 men only n = 6,897 Age 51–59 years Mean age at baseline DM 55.5 (2.1) No-DM 55.3 (2.2)	Prevalent non-differentiated DM (known)	Self-reported DM n = 232	Non-fatal AMI or CHD death	Hospital records and death certificate	AMI at baseline	N/A
Saeed, M 2022 Norway [48]	1973–2017 prospective 20Y CumI 40Y CumI	Nationwide data of people with type 1 DM and matched controls from Norway n = 76,442 Age without limitation Mean age at end of follow-up (range) DM 31.8 (0.8–59.1) Controls 32.1 (1.42–59.5)	Prevalent type 1 DM	People with childhood-onset type 1 DM (age at diagnosis DM ≤ 14 Y) from the Norwegian Childhood Diabetes Registry (NCDR) Mean DM duration = 22.4 years n = 7,086	Non-fatal AMI: discharge diagnosis Fatal AMI ICD-9 410; ICD-10 I21, I22	Non-fatal AMI: Hospitalization with AMI based on CVDNOR Project during 1994–2008 and on the Norwegian Patient Registry from 2008 to 2017 Fatal AMI: The Norwegian Cause of Death Registry	People with CHD at baseline were not excluded	CumI + HR +
Schramm, T 2008 Denmark [49]	1997–2002 retrospective5Y CumI	Residents of Denmark, n = 3,194,898 Age ≥ 30 years Mean age at baseline Women DM 65.0 (15.1) No-DM 53.4 (16.1) Men DM 60.4 (14.2) No-DM 50.0 (14.5)	Prevalent non-differentiated DM (known)	People with glucose-lowering drug from the National Prescription Registry n = 65,382	AMI I21-I22 or CHD death I20-I25 and I46	Non-fatal AMI: National Patient Registry Fatal AMI: Central Population Register, National Registry of Causes of Death	AMI at baseline	N/A

*AMI* Acute myocardial infarction, *CHD* Coronary heart disease, *CumI* Cumulative incidence, *CVD* Cardiovascular disease, *DM* Diabetes mellitus, *ICD* International classification of diseases, *ICH* Ischemic heart disease, *IFG* Impaired fasting glycaemia, *IGT* Impaired glucose tolerance, *IR* Incidence rate, *HR* Hazard ratio, *LEA* Lower extremity amputation, *N/A* Not available, *PAD* Peripheral arterial disease, *RR* Relative risk

**Table 2:** Incidence of acute myocardial infarction in people with and without diabetes as well as relative risks or hazard ratios

Study	Number of AMI (Non-DM/DM population	IR in DM population (per 100,000 PY)	IR in non-DM population (per 100,000 PY)	Estimates comparing people with and without DM (95% CI)
**I. Incidence rates**				
**Incident type 2 DM**				
Wright, A [44] UK 2006–2015	Non-DM n = 6,111 Women n = 2,524 Men n = 3,587 DM n = 2,697 Women n = 1,118 Men n = 1,579	N/A	N/A	Multiple-adjusted HR^*^ Women 1.55 (1.44–1.67) Men 1.39 (1.30–1.48)
**Incident DM without distinguishing between types**				
Barengo, N [12] Finland Cohort 1:1972–1977 + 10 years follow-up Cohort 2: 1982–1987 + 10 years follow-up	NA	N/A	N/A	Age-sex stratified RR Cohort 1 Women All 6.36 25–49 7.75 50–64 3.43 Men All 3.53 25–49 3.72 50–64 2.28 Cohort 2 Women All 6.50 25–49 11.5 50–64 3.05 Men All 3.18 25–49 3.12 50–64 2.00
de Jong, M [5] UK 2006–2018	Non-DM n = 5,192 Women n = 1,533 Men n = 3,659 Pre-diabetes n = 1,209 Women n = 417 Men n = 792 DM n = 110 Women n = 26 Men n = 84	Multiple-adjusted IR^†^ Women 143 (84–201) Men 389 (302–476)	Multiple-adjusted IR^†^ Non-DM: Women 87 (82–92) Men 254 (245–263) Pre-diabetes: Women 109 (98–12) Men 297 (275–319)	Age-adjusted HR Women 2.55 (1.73–3.76) Men 2.03 (1.63–2.52)
**Prevalent type 1 DM**				
Dybjer, E [28] Sweden 1959/1961 −2018	Non-DM men n = 63 DM men n = 33	NA	NA	Age-adjusted HR Only men 4.17 (2.72–6.37)
Matuleviciene, V [35] Sweden 1998–2011	Non-DM n = 1,925 Women n = 533 Men n = 1,392 DM n = 1,500 Women n = 694 Men n = 806	N/A	N/A	Age-(sex)-adjusted HR 4.38 (4.1–4.69) Women 18–49 19.15 (13.57–27.03) 50–64 8.56 (7.13–10.27) 65 + 5.21 (4.40–6.17) Men 18–49 4.65 (3.83–5.65) 50–64 3.04 (2.68–3.44) 65 + 2.73 (2.34–3.19)
Millett, E [36] UK 2006–2016	NA	Multiple-adjusted IR^‡^ Women 649.2 (358.8–939.6) Men 619.2 (351.7–886.7)	Multiple-adjusted IR^‡^ Women 79.5 (75.0–84.0) Men 220.6 (212.7–228.4)	Multiple-adjusted HR^‡^ Women 8.18 (5.20–12.86) Men 2.81 (1.82–4.33)
**Prevalent type 2 DM**				
Ballotari, P [27] Italy 2012–2014	Non-DM n = 2,303 Women n = 713 Men n = 1,590 DM n = 700 Women n = 241 Men n = 459	Age-adjusted IR Women 475.8 Men 780.2	Age-adjusted IR Women 161.3 Men 390.4	RR adjusted for age and foreign status Total 2.00 (1.83–2.18) Women 2.58 (2.22–3.00) Men 1.78 (1.60–2.00)
Fujishima, M [30] Japan 1988–1993	Total (non-DM and DM) n = 28	Age- and sex-adjusted IR 500	Age- and sex-adjusted IR 160	Age- and sex-adjusted RR 2.6 (1.05–6.4)
Juutilainen, A [32] Finland 1982–1996	Non-DM n = 95 Women n = 16 Men n = 79 DM n = 277 Women n = 126 Men n = 151	N/A	N/A	HR adjusted for age and location Women 14.37 (8.43–24.47) Men 2.92 (2.21–3.86)
Millett, E [36] UK 2006–2016	NA	Multiple-adjusted IR^‡^ Women 156.3 (126.4–186.2) Men 294.1 (259.2–329.1)	Multiple-adjusted IR^‡^ Women 79.5 (75.0–84.0) Men 220.6 (212.7–228.4)	Multiple-adjusted HR^‡^ Women 1.96 (1.60–2.41) Men 1.33 (1.18–1.51)
Read, SH [4] UK 2006–2015	Non-DM n = 105,536 Women n = 39,925 Men n = 65,611‬ DM n = 24,390 Women n = 9,297‬ Men n = 15,093‬	NA	NA	RR adjusted for age, calendar year and deprivation Women 2.32 (2.15–2.51) Men 1.86 (1.74–1.98)
Tancredi, M [41] Sweden 1998–2012	Non-DM n = 115,172 Women n = 43,992 Men n = 71,180 DM n = 36,124 Women n = 14,781 Men n = 21,343	N/A	N/A	Unadjusted RR Total 1.68 (1.66–1.70) Women 1.79 (1.76–1.82) Men 1.61 (1.58–1.63) Age-adjusted HR Total 1.67 (1.65–1.69) Women < 55 5.09 (4.35–5.95) 55–64 3.05 (2.83–3.29) 65–74 2.37 (2.27–2.48) ≥ 75 1.61 (1.57–1.64) Men < 55 2.93 (2.71–3.17) 55–64 1.97 (1.89–2.05) 65–74 1.64 (1.60–1.69) ≥ 75 1.41 (1.38–1.44)
**Prevalent DM without distinguishing between types**				
Barengo, N [12] Finland Cohort 1: 1972–1977 + 10 years follow-up Cohort 2: 1982–1987 + 10 years follow-up	NA	N/A	N/A	Age-sex-stratified RR Cohort 1 Women All 2.33 25–49 2.00 50–64 1.84 Men All 1.67 25–49 1.75 50–64 1.32 Cohort 2 Women All 3.42 25–49 3.86 50–64 3.18 Men: All 1.37 25–49 1.28 50–64 1.45
Carson, A [13] USA ARIC study 1987–1996 REGARD study 2003–2009	NA	IR adjusted for age, sex, ethnicity ARIC 1110 (930–1320) REGARD 540 (420–700)	IR adjusted for age, sex, ethnicity ARIC 390 (350–440) REGARD 220 (180–270)	HR adjusted for age, sex, ethnicity ARIC 2.83 (2.29–3.49) REGARD 2.44 (1.78–3.34)
de Jong, M [5] United Kingdom 2006–2018	Non-DM n = 5,192 Women n = 1,533 Men n = 3,659 Pre-diabetes n = 1,209 Women n = 417 Men n = 792 DM n = 805 Women n = 221 Men n = 584	Multiple-adjusted IR^†^ Women 204 (171–236) Men 461 (414–508)	Multiple-adjusted IR^†^ Non-DM Women 87 (82–92) Men 254 (245–263) Pre-diabetes Women 109 (98–12) Men 297 (275–319)	Age-adjusted HR Women 3.02 (2.62–3.48) Men 1.85 (1.69–2.02)
Folsom, A [29] USA 1987–1995	Non-DM n = 229 Women n = 63 Men n = 166 DM n = 76 Women n = 33 Men n = 43	IR adjusted for age, sex, study center Women 690 Men 1630	IR adjusted for age, sex, study center Women 170 Men 680	Multiple-adjusted RR^§^ Total Women 3.45 (2.16–5.50) Men 2.52 (1.78–3.56) Black Women 3.1 (1.6–6.1) Men 1.6 (0.83–3.3) Other ethnicities Women 3.9 (2.0–7.4) Men 3.0 (2.0–4.5)
Hyvarinen, M [31] Finland Finland 1987–2006 Sweden 1986–2004	Non-DM n = 438 Women n = 145 Men n = 293 DM n = 92 Women n = 37 Men n = 55	NA	NA	Multiple-adjusted HR^||^ Women 2.48 (1.69–3.65) Men 2.09 (1.55–2.82)
Icks, A [15] Germany 1985–2006	Non-DM n = 10,446 Women n = 2,752 Men n = 7,694 DM n = 4,132 Women n = 1,497 Men n = 2,635	NA	NA	RR adjusted for sex and calendar year Women 6.38 (5.36–7.58) Men 3.30 (2.74–3.95)
Liu, F [33] China 2000–2008	Non-DM n = 80 IFG n = 34 DM n = 20	N/A	N/A	Age-adjusted HR 2.62 (1.59–4.33)
Lundberg, V [34] 1997 Sweden 1989–1993	Non-DM n = 2,543 Women n = 491 Men n = 2,052 DM n = 488 Women n = 125 Men n = 363	N/A	N/A	RR from crude IR; CI from Epi-Info computer program Women 5.0 (3.9–6.3) Men 2.9 (2.6–3.4)
Moe, B [37] Norwegen 1995–2008	Non-DM n = 1,715 DM n = 172	NA	NA	HR adjusted for age and birth cohort (5-year strata) Women 2.76 (2.17–3.51) Men 1.49 (1.20–1.86)
Oğuz, A [38] Türkiye 2008–2020	Non-DM n = 99 DM n = 42	N/A	N/A	Age-sex adjusted HR 2.50 (1.73–3.62)
Saito, I [40] Japan 1990–2006	Non-DM n = 182 Borderline n = 55 DM n = 29	Age-adjusted IR Women 102 Men 206	Age-adjusted IR Non-DM Women 30 Men 100 Borderline Women 29 Men 137	HR adjusted for sex, age, blood status (fasting/non-fasting) 3.05 (2.03–4.59)
Vimalananda, V [42] USA 1989–2002	Non-DM n = 998 White women n = 449 White men n = 431 Black women n = 65 Black men n = 53 DM n = 248 White women n = 82 White men n = 104 Black women n = 37 Black men n = 25	NA	NA	HR adjusted for age and clinic White Women 2.13 (1.68–2.70) Men 1.83 (1.48–2.27) Black Women 2.38 (1.59–3.57) Men 1.54 (0.96–2.49)
Wannamethee, S [43] UK 1998–2008	Only men Non-DM n = 229 DM n = 54 Early-onset diabetes n = 18 Late-onset diabetes n = 36	NA	NA	Age-adjusted HR Only men Early-onset diabetes 2.93 (1.81–4.74) Late-onset diabetes 1.70 (1.20–2.42)
**II. Cumulative Incidence**				
**Incident type 2 DM**				
Nørgaard, C [46] Denmark 1997–2014	Non-DM n = 36,762 DM n = 5,542	5Y CumI 1997–2002 4.1% (4.0–4.2) 2003–2008 2.5% (2.4–2.6) 2009–2014 1.9% (1.8–1.9)	5Y CumI 1997–2002 2.4% (2.3–2.4) 2003–2008 1.8% (1.8–1.8) 2009–2014 1.5% (1.4–1.5)	Multiple-adjusted HR^¶^ 1997–2002 1.89 (1.83–1.94) 2003–2008 1.46 (1.41–1.51] 2009–2014 1.31 (1.25–1.37)
**Prevalent type 1 DM**				
Saeed, M [48] Norway 1973–2017	Non-DM n = 193 DM n = 170	20Y CumI 0.4% (0.3–0.6) 40Y CumI 8.0% (6.8–9.4)	20Y CumI 0.04% (0.02–0.06) 40Y CumI 1.13% (0.96–1.31)	HR adjusted for education and origin HR 9.05 (7.18–11.41)
**Prevalent type 2 DM**				
Haffner, S [45] Finland 1982–1989	NA	7Y CumI 20.2%	7Y CumI 3.5%	NA
**Prevalent DM without distinguishing between types**				
Rosengren, A [47] Sweden 1974–1983	Non-DM n = 330 DM n = 31	7Y CumI 13.4%	7Y CumI 5.0%	NA
Schramm, T [49] Denmark 1997–2002	Non-DM n = 54,018 Women n = 21,787 Men n = 32,231 DM n = 4,634 Women n = 2,168 Men n = 2,466	5Y CumI Women 10.6% Men 11.1%	5Y CumI Women 2.2% Men 3.0%	Age-adjusted HR Women 2.85 (2.75–2.96) Men 2.32 (2.25–2.40)

*AMI* Acute myocardial infarction,* CumI* Cumulative incidence,* DM* Diabetes mellitus, *HR* Hazard ratio,* IFG* Impaired fasting glycemia,* IGT* Impaired glucose tolerance,* IR* Incidence rate, *Non-DM* Population without diabetes,* N/A* Not available,* PY* Person-years, *RR* Relative risk

^*^HR adjusted for baseline calendar year, age, GP practice, ethnicity, and disadvantage

^†^IR adjusted for age, smoking (never, former, current), BMI, systolic blood pressure, lipid-lowering medications, cholesterol, antihypertensive medications, the Townsend social deprivation score, and interaction terms between each variable and sex

^‡^IR and HR adjusted for age, systolic blood pressure, and socioeconomic status

^§^RR adjusted for age, race, study center, smoking, ethanol drinking, education, sport index, hormone replacement only in women

||HR adjusted for study, hypertension status, BMI, serum total cholesterol, HDL cholesterol, smoking

^¶^HR adjusted for age, sex, calendar year, history of hypertension, chronic kidney disease

**Table 3:** Time trends in the incidence of acute myocardial infarction

Study	Time trend of estimates (95% CI) in DM population	Time trend of estimates (95% CI) in non-DM population	Time trend of estimates comparing people with and without DM (95% CI)
**I. Incident rates**			
**Incident type 2 DM**			
Wright, A [44] UK 2006–2015	N/A	N/A	RR^†^ No significant change Women: 2007–2010 1.64 (1.48–1.82) 2011–2013 1.52 (1.24–1.86) Men: 2007–2010 1.34 (1.23–1.47) 2011–2013 1.56 (1.33–1.82)
**Incident DM without distinguishing between types**			
Barengo, N [12] Finland Cohort 1: 1972–1977 + 10 years follow-up Cohort 2: 1982–1987 + 10 years follow-up	IR cohort 1/IR cohort 2 Women All No sign. change 1.4 (0.8–2.4) 25–49 No sign. change 2.2 (0.5–20.1) 50–64 No sign. change 1.4 (0.8–2.5) Men All Decreasing T^**^ 1.5 (1.0–2.5) 25–49 Decreasing T^**^ 2.8 (1.0–10.9) 50–64 Decreasing T^**^ 1.6 (1.0–2.8)	IR cohort 1/IR cohort 2 Women All Decrease 1.4 (1.2–1.7) 25–49 Decrease 3.3 (2.0–5.9) 50–64 Decreasing T^**^ 1.2 (1.0–1.5) Men All Decrease 1.4 (1.2–1.6) 25–49 Decrease 2.3 (1.8–2.9) 50–64: Decreasing T^**‡^	Descriptive comparison of age-sex-stratified RR Women All Increasing T^*^ Cohort 1 6.36 Cohort 2 6.50 25–49 Increasing T^*^ Cohort 1 7.75 Cohort 2 11.5 50–64 Decreasing T^**^ Cohort 1 3.43 Cohort 2 3.05 Men All Decreasing T^**^ Cohort 1 3.53 Cohort 2 3.18 25–49 Decreasing T^**^ Cohort 1 3.72 Cohort 2 3.12 50–64 Decreasing T^**^ Cohort 1 2.28 Cohort 2 2.00
**Prevalent type 1 DM**			
Matuleviciene, V [35] Sweden 1998–2011	N/A	N/A	Age-sex-adjusted HR Descriptive decrease until 2005 4.80 (4.26–5.42) since 2005 3.63 (3.33–3.95)
**Prevalent type 2 DM**			
Read, SH [4] UK 2006–2015	RR adjusted for age and deprivation (for whole population: people with and without diabetes) Decrease 2% (1.73–2.26) per year		RR adjusted for age and deprivation No change 1.01 (1.00–1.02)
Tancredi, M [41] Sweden 1998–2012	N/A	N/A	Age-sex-adjusted HR Decrease (p < 0.001) until 2005 1.57 (1.52–1.62) since 2005 1.39 (1.37–1.41)
Carson, A [13] USA Two prospective cohort studies ARIC study 1987–1996, REGARD study 2003–2009	HR adjusted for age, sex, ethnicity Total Decrease 0.53 (0.38–0.73) Women No change 1.06 (0.61–1.85) Men Decrease 0.48 (0.28–0.81)	HR adjusted for age, sex, ethnicity Decrease 0.60 (0.48–0.76)	HR adjusted for age, sex, ethnicity No change (p = 0.39)
Rautio, A [39] Sweden 1989–2000	Change per year in % based on age-adjusted RR Women No sign. change −3,1% (−7,7 to 1,3) Men No change 0,5% (−2,1 to 3,1)	Change per year in % based on age-adjusted RR Women No change −0,35% (−2,3 to 1,6) Men Decrease −4,1% (−5,1 to −3,0)	Age-adjusted RR Women no sign. change Men no sign. change
**Prevalent DM without distinguishing between types**			
Barengo, N [12] Finland Cohort 1: 1972–1977 + 10 years follow-up Cohort 2: 1982–1987 + 10 years follow-up	IRR cohort 1/cohort 2 Women All No change 1.0 (0.6–1.7) 25–49 No sign. change 1.6 (0.4–7.4) 50–64 No sign. change 0.7 (0.4–1.3) Men All Decreasing T^**^ 1.7 (1.0–2.8) 25–49 Decrease 3.2 (1.3–9.6) 50–64 No change 1.1 (0.6–2.0)	RR cohort 1/cohort 2 Women All Decrease 1.4 (1.2–1.7) 25–49 Decrease 3.3 (2.0–5.6) 50–64 Decreasing T^**^ 1.3 (1.0–1.6) Men All Decrease 1.4 (1.2–1.6) 25–49 Decrease 2.3 (1.8–2.9) 50–64 Decreasing T^**^ 1.2 (1.0–1.4)	Descriptive comparison of age-sex stratified RR Women All Increasing T^*^ Cohort 1 2.33 Cohort 2 3.42 25–49 Increasing T^*^ Cohort 1 2.00 Cohort 2 3.86 50–64 Increasing T^*^ Cohort 1 1.84 Cohort 2 3.18 Men All Decreasing T^**^ Cohort 1 1.67 Cohort 2 1.37 25–49 Decreasing T^**^ Cohort 1 1.75 Cohort 2 1.28 50–64 Increasing T^*^ Cohort 1 1.32 Cohort 2 1.45
Icks, A [15] Germany 1985–2006	Age-adjusted RR per calendar year Women Decrease 0.99 (0.98–0.99) Men Increase 1.01 (1.00–1.02)	Age-adjusted RR per calendar year Women Decrease 0.99 (0.98–0.99) Men Decrease 0.98 (0.98–0.99)	Age-adjusted RR per calendar year Women No change 1.00 (0.99–1.01) Men Increase 1.03 (1.01–1.04)
**II. Cumulative Incidence**			
**Incident type 2 DM**			
Nørgaard, C [46] Denmark 1997–2014	5Y CumI Decrease 1997–2002 4.1% (4.0–4.2) 2003–2008 2.5% (2.4–2.6) 2009–2014 1.9% (1.8–1.9)	5Y CumI Decrease 1997–2002 2.4% (2.3–2.4) 2003–2008 1.8% (1.8–1.8) 2009–2014 1.5% (1.4–1.5)	Multiple-adjusted HR^§^ Decrease: 1997–2002 1.89 (1.83–1.94) 2003–2008 1.46 (1.41–1.51) 2009–2014 1.31 (1.25–1.37)
**Prevalent type 1 DM**			
Saeed, M [48] Norway 1973–2017	HR^†^ adjusted for age, sex, education, origin No change 0.997 (0.967–1.028)	HR^†^ adjusted for sex, education, origin Increase 1.05 (1.02–1.09)	HR^†^ adjusted for education and origin No significant change

*AMI* Acute myocardial infarction, *CumI* Cumulative incidence, *DM* Diabetes mellitus, *IR* Incidence rate, *HR* Hazard ratio, *N/A* Not available, *RR* Relative risk

^*^Increasing tendency

^**^Decreasing tendency

^†^Time trend for calendar year of DM diagnosis

^‡^IR cohort 2 < than IR cohort 1 (22.3 (95% CI 19.5–25.3) vs. 26.9 (95% CI 24.8–29.1))

^§^HR adjusted for age, sex, calendar year, history of hypertension, chronic kidney disease

##### Outcome

The main outcome of the included studies was the incidence of AMI in people with and without diabetes. The included studies needed to report both non-fatal and fatal AMI.

##### Epidemiological measures

Studies were included if they reported either (a) incidence rate (IR) or cumulative incidence (CumI) in people with diabetes and people without diabetes, or (b) relative risk (RR) or hazard ratio (HR) comparing incidence rates in these populations (hereafter referred to as “estimates”), or (c) corresponding time trends. To ensure an appropriate comparison between people with diabetes and those without, the incidence had to be reported at least as an age-adjusted or standardized estimate.

Original full-text articles were included if they fulfilled the inclusion criteria concerning type of study (population-based cohort studies only), type of population (with and without diabetes), main outcome (fatal and non-fatal AMI), and appropriate epidemiological measures regardless of the time period and year of study publication, diabetes type, age and sex distribution, or ethnicity. Table S2 summarizing inclusion and exclusion criteria is provided in the supplementary information.

### Exclusion criteria

Studies were excluded if (a) they reported only the incidence of non-fatal AMI or only the incidence of fatal AMI; (b) they solely reported the incidence of AMI in people with diabetes but not in those without diabetes; (c) the incidence rates were reported in relation to the total population (with and without diabetes) and did not exclusively use the population with diabetes as the at-risk population; (d) only crude incidence rates (calculated by dividing the total number of cases in a given time period by the total number of persons in the population) were reported; and (e) they were published in a language other than English.

### Study selection process

Titles and abstracts of all references were independently screened by two authors (MN, EU) to identify original research articles for AMI incidence according to the predefined inclusion criteria. Two review authors (MN, EU) then independently screened the full-text articles. Disagreements regarding the inclusion or exclusion of studies were resolved in discussion with a third review author (AI or TK or HC).

### Methodological quality assessment and data extraction

Two authors (MN, EU) independently conducted a critical appraisal of the studies to evaluate methodological quality and potential risk of bias of the eligible studies using the modified checklist adapted from the Methodological Evaluation of Observation Research (MORE), the Scottish Intercollegiate Guidelines Network (SIGN), and the Cochrane Consumers and Communication Review Group’s Study Quality Guide. We assessed the following components that could potentially bias estimates of AMI: study population characteristics (nationwide vs. regional/local studies), definition of population with diabetes (medical diagnosis of diabetes vs. self-reported diabetes), definition of outcome AMI (clear definition of AMI vs. unclear definition), number of AMI cases (sufficient number > 10 cases vs. very small number ≤ 10 cases), precision and consistency of results (narrow 95% confidence interval (95% CI) vs. wide 95% CI or absence of 95% CI*),* and reporting of time trend (use of multivariate regression models vs. simple description without appropriate statistical models). On that basis, studies were classified as having a low, high, or unclear risk of bias according to the Cochrane recommendations. Detailed information regarding bias assessment across the included studies is provided in Table S3 in the supplementary information.

We developed a data extraction sheet based on the Cochrane Consumers and Communication Review Group’s data extraction template, which was initially tested on five randomly selected included papers. One author extracted data (see data items below) from the included articles; the other author checked the extracted data. Disagreements were discussed and if agreement could not be reached, a third author was consulted to resolve discrepancies.

We extracted the following information from each included article: first author, publication year, country, study period and study design, study population, diabetes type, characteristics of people with diabetes, definition of fatal and non-fatal AMI, data source of AMI, presence or absence of comorbidity relevant to AMI incidence (e.g., a history of CHD or CVD in the population at risk), absolute numbers and incidence (incidence rate or cumulative incidence) of AMI, estimates comparing the incidence of AMI in people with and without diabetes, and time trends when available. We recalculated the reported IR per 100,000 person-years if not originally reported as such. Given the considerable heterogeneity of the included studies, no meta-analysis of results was conducted. In line with our systematic review protocol, heterogeneity was judged with regard to content and not by performing prior statistical tests for homogeneity [25]. Data are presented descriptively in tables as age-sex adjusted, multiple adjusted, or standardized estimates; 95% CIs were provided if available.

## Results

**Fig. 1:**
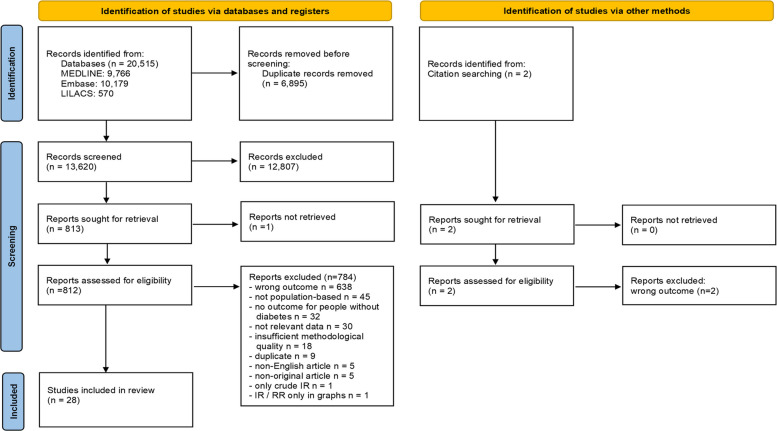
PRISMA 2020 flow diagram

We initially retrieved 20,515 citations from the databases searches, of which 6895 duplicate records were removed. A total of 13,620 records were screened and 12,807 of these were excluded. Two additional articles were identified by checking the reference lists of selected papers and one report could not be retrieved. Of 814 full texts assessed for eligibility, we excluded 786 articles, of which 18 studies had insufficient methodological quality with a high risk of bias. The main reasons for the exclusion of these 18 studies were an unclear definition of AMI, an unclear description of statistical methods, and a very small number of AMI cases (≤ 10 cases). We thus ultimately included 28 studies in this review [4, 5, 12, 13, 15, 27–49]. The selection procedure is summarized in Fig. 1.

### Study characteristics

The majority of studies were prospective, while five studies were retrospective [4, 31, 44, 46, 49], population-based longitudinal studies. Most of the studies were conducted in Europe (21 of 28), whereas three studies were from the USA [13, 29, 42] and four studies from Asia [30, 33, 38, 40]. In most of the studies, the age of the study population ranged between 25 and 74 years. While the majority of the studies analyzed the incidence of AMI in both men and women, three studies were restricted to men only [28, 43, 47]*.* The detailed characteristics of the studies are presented in Table 1.

### Epidemiological measures

#### Incidence rate of AMI in people with and without diabetes

We identified seven studies that reported the age- or age–sex–adjusted IR of AMI in people with and without diabetes [5, 13, 27, 29, 30, 36, 40], including one study in people with incident diabetes [5] (Table 2).

Among people with diabetes, the IR of AMI varied in women from 102 per 100,000 PY in Japan, 1990–2006 [40] to 690 per 100,000 PY in the USA, 1987–1995 [29] and in men from 206 to 1630 per 100,000 PY in the same studies. The IR in people with incident diabetes (women 143, men 389 per 100,000 PY) was lower than the IR in people with prevalent diabetes (women 204, men 461 per 100,000 PY) [5].

Among people without diabetes, the IR of AMI in women ranged from 30 per 100,000 PY in Japan, 1990–2006 [40] to 170 per 100,000 PY in the USA, 1987–1995 [29] and in men from 100 to 680 per 100,000 PY in the same studies. Thus, most studies showed a higher IR of AMI in men than in women, irrespective of diabetes status. However, one study in people with type 1 diabetes did not observe that difference: women 649.2 (358.8–939.6) and men 619.2 (351.7–886.7) [36].

#### Cumulative incidence

Five studies analyzed the CumI of AMI [45–49] (Table 2). The CumI in people with diabetes ranged from 0.4% after 20 years of diabetes duration among people with type 1 diabetes in Norway, 1973–2017 [48] to 20.2% after 7 years of follow-up among people with type 2 diabetes in Finland, 1982–1989 [45]. Among people without diabetes, CumI varied between 0.04% during the first 20 years of follow-up in Norway, 1973–2017 [48] and 5% during 7 years of follow-up in Sweden, 1974–1983 [47].

#### Estimates comparing the incidence of AMI in people with and without diabetes

We identified 25 studies that reported estimates comparing people with and without diabetes (HR or RR). Two studies showed separate data for cohorts with incident and prevalent diabetes [5, 12], and one study presented separate data for cohorts with type 1 and type 2 diabetes [36] (Table 2).

The estimates comparing people with and without diabetes ranged from 1.55 (1.44–1.67) in the UK, 2006–2015 [44] to 14.37 (8.43–24.47) in Finland, 1982–1996 [32] in women, and from 1.33 (1.18–1.51) in the UK, 2006–2016 [36] to 4.17 (2.72–6.37) in Sweden, 1959–2018 [28] in men (Supplementary Fig. S1 and Fig. S2).

One US study found in both sexes similar estimates comparing people with and without diabetes, with no significant difference observed between Black and White people [42]. Another US study also found no discrepancies regarding ethnicity among women. However, some lower estimates were found in Black men compared with men of other ethnicities [29].

### Time trends

#### Incidence rate of AMI

**Fig. 2:**
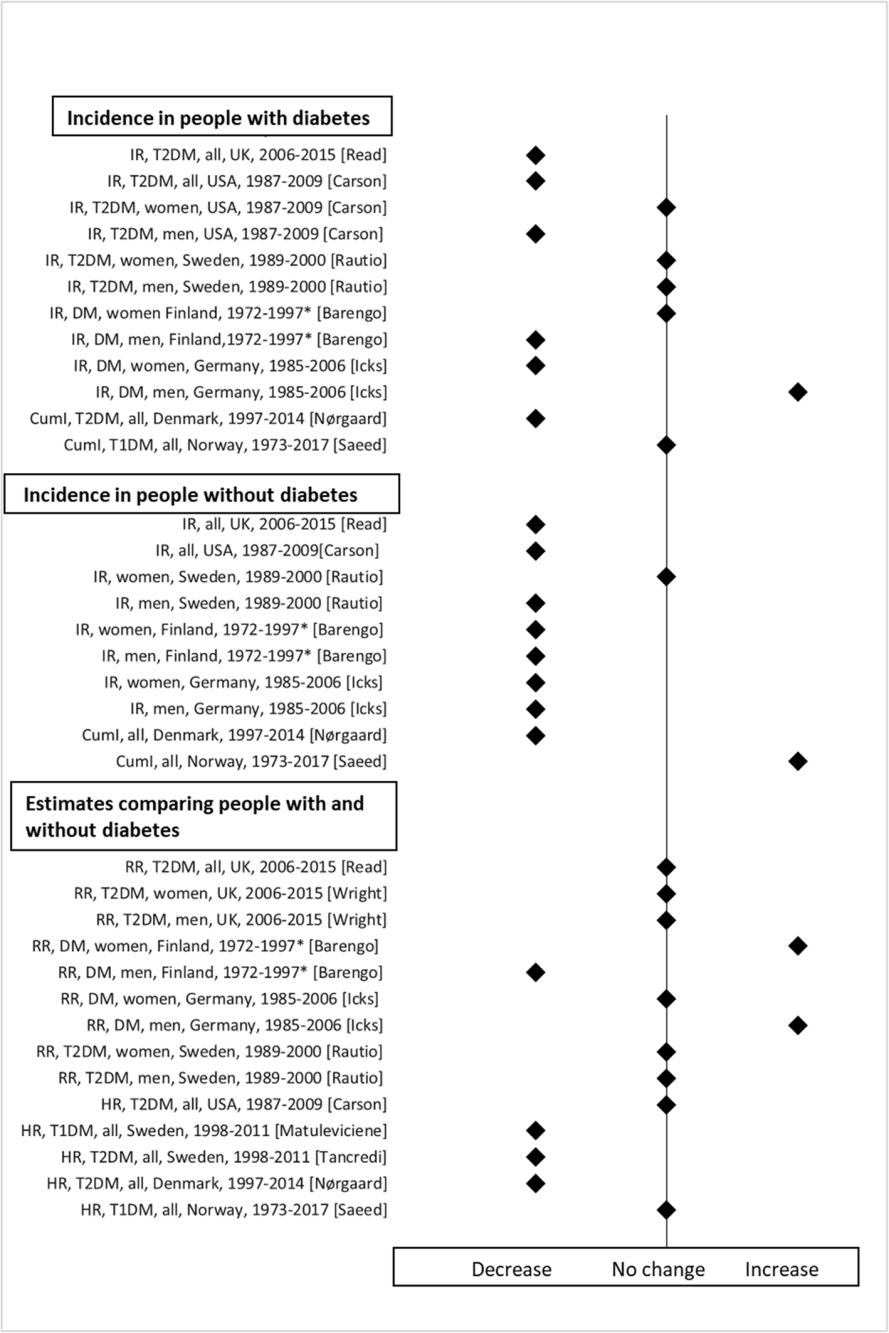
Time trends in the incidence of AMI. *IR* incidence rate, *CumI* cumulative incidence, *DM* diabetes without distinguishing between types, *T1DM* type 1 diabetes, *T2DM* type 2 diabetes, *RR* relative risk, *HR* hazard ratio. ^*^This study found similar time trends in cohorts with prevalent as well as incident diabetes

Five studies analyzed time trends of the IR of AMI [4, 12, 13, 15, 39], including one study examining time trends of the IR among people with incident diabetes [12]. Almost all studies reported a decrease in the IR of AMI in people without diabetes, while studies of people with diabetes showed contradictory results (Table 3 and Fig. 2). A recent Scottish study found a decreased IR in people with and without diabetes between 2006 and 2015 with consistent results in men and women [4]. A US study investigating changes in the IR between two study periods 1987/1996 and 2003/2006 found a decreased IR in men with diabetes and in people without diabetes. However, the IR in women with diabetes remained constant [13]. A Finnish study also observed similar trends between 1972 and 1997 [12]. A Swedish study from 1989 to 2000 reported a significant decrease in the IR only in men without diabetes, with no significant changes in other sub-groups [39]. However, a German study reported a significant increase in the IR in men with diabetes, as well as a decrease among women with diabetes and among people without diabetes between 1985 and 2006 [15].

#### Cumulative incidence

Two studies analyzed time trends of the CumI [46, 48]. The Danish study observed a decline of the CumI both in people with incident type 2 diabetes and in those without diabetes during 1997–2014 [46]. In contrast, the Norwegian study reported no change of the CumI in people with prevalent type 1 diabetes and an increased time trend in matched controls during 1973–2017 [48] (Table 3 and Fig. 2).

#### Estimates comparing the incidence of AMI in people with and without diabetes

Ten studies reported time trends of estimates (HR/RR), comparing people with and without diabetes, including three studies analysing time trend in people with incident diabetes [12, 44, 46]. The results of these studies were contradictory (Table 3 and Fig. 2). Two studies from Sweden during the period 1998–2012 [35, 41] as well as a study from Denmark during the period 1997–2014 [46] found decreased time trends of HR. A Finnish study reported decreased RR in men but increased RR in women in both prevalent and incident cohorts between 1972 and 1997 [12]. In contrast, a German study reported increased RR in men and no changes in women between 1985 and 2006 [15]. Five studies found no significant changes [4, 13, 39, 44, 48].

### Gender and geographical differences

**Fig. 3:**
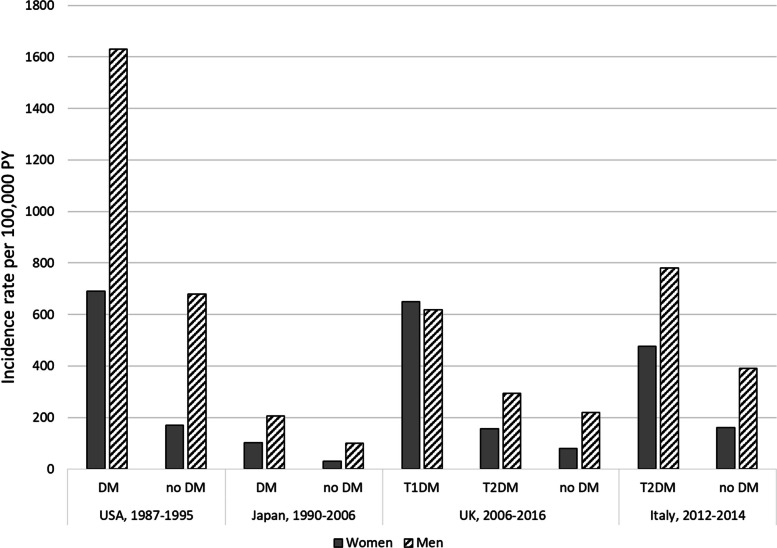
Incidence rate of AMI in people with and without diabetes. *DM* diabetes without distinguishing between types, *no DM* population without diabetes, *T1DM* type 1 diabetes, *T2DM* type 2 diabetes

The majority of studies found a higher risk of AMI in men than in women, with the exception of one study from the UK among people with type 1 diabetes [36] (Fig. 3). However, the estimates comparing incidences in people with and without diabetes were significantly higher in women (Supplementary Fig. S1 and Fig. S2). A comparison of studies showed a lower incidence in Japan [40] and a higher incidence in the USA (Fig. 3), where a percentage of the study population was of African-American origin [13, 29], which is a known risk factor for CHD [50, 51]. With regard to estimates comparing people with and without diabetes stratified by ethnicity, one of the two available studies reported lower estimates in African-American men than in men of other ethnicities [29]. No other changes were reported [29, 42].

### Type 1 and type 2 diabetes

The reported incidence of AMI was higher in people with type 1 diabetes than in those with type 2 diabetes (Fig. 3). This difference was approximately twofold in men and fourfold in women [36]. The HRs comparing incidence in people with and without diabetes were also higher in people with type 1 diabetes [28, 35, 36, 48] with particularly notable differences among women [35, 36, 41]. While studies in people with type 2 diabetes found higher IR in men than in women, no sex difference was observed in people with type 1 diabetes (Fig. 3) [36].

### Risk of bias in studies reviewed

#### Population with diabetes

A correct analysis of the incidence of AMI among people with diabetes relies predominantly on an accurate estimation of the number of people with diabetes in the background population, which serves as a denominator for the incidence. Some studies defined the population with diabetes using self-reported patient data, which might lead to misclassification due to underreporting of diabetes. Only a few studies used data from national diabetes registers or from national prescription/reimbursement registers. The remaining studies employed various sources to identify people with diabetes. Consequently, the population with diabetes was defined in a very broad manner: people with known diabetes, people with known treated diabetes who were identified by prescription of glucose-lowering medication, and people with both known and unknown diabetes, based on HbA1c-values or blood glucose measurements.

Furthermore, the diagnostic criteria of diabetes have been changed several times over the last 30 years. The first change was between 1997 and 1999, lowering the fasting plasma glucose concentration threshold from 140 mg/dl (7.8 mmol/l) to 126 mg/dl (7.0 mmol/l) [52]. The diagnostic criteria of diabetes were subsequently revised between 2009 and 2011 and included HbA1c testing with a cut point ≥ 6.5% as the preferred method for diagnosis [53]. This revised definition of diabetes could lead to a higher estimate of the number of people with diabetes, thereby including a higher percentage of those in the earlier or less severe stages. This should also be taken into consideration, particularly for the time trend analyses.

#### Definition of AMI

In interpreting the results, it is very important to consider the various aspects pertaining to the recording of AMI in the studies under review. Firstly, a broad definition of AMI was used. Several studies recorded non-fatal AMI and fatal CHD whereas other studies recorded non-fatal and fatal AMI. Secondly, the diagnostic criteria for AMI have changed over the past few decades. Modern laboratory methods based on troponin markers and later high-sensitivity troponin markers have been in use since 2000 [54–57]. Thirdly, some recurrent AMIs might have been misclassified as an incident AMI, especially in retrospective studies.

## Discussion

This systematic review including 28 studies revealed that the incidence of AMI remained higher in people with diabetes than those without. Almost all studies found a higher incidence of AMI in men than in women, irrespective of diabetes status. However, women with diabetes exhibited a markedly elevated risk in comparison to women without diabetes. Nearly all studies investigating time trends reported a declining incidence of AMI in people without diabetes, although the time trends in people with diabetes were less consistent. Consequently, the time trends of estimates comparing people with and without diabetes differed. Study results varied largely, which can be attributed, at least in part, to the methodological differences between the included studies. As previously outlined, there was a high degree of heterogeneity of the included studies regarding AMI definition, diabetes population, statistical approach, and study period. This heterogeneity was further compounded by the various healthcare systems, which differ, for example, in terms of access to care, treatment standards, and management of risk factors. Therefore, no quantitative data synthesis was performed.

### Estimates comparing people with and without diabetes

The estimates (RR or HR) comparing incidences of AMI in people with and without diabetes remained high. In particular, women with diabetes exhibited a significantly greater excess risk than women without diabetes, while this excess risk was substantially less pronounced in men. Our results are in line with findings from a systematic review regarding sex disparities in incident CHD, which found poorer outcomes in women with diabetes than in men with diabetes, in each case compared to counterparts without diabetes. The ratio comparing the RR for women to the RR for men was 1.44 (95% CI 1.28–1.63) [18]. One hypothesis is that a combination of various factors, including biological factors (e.g., (gluco) metabolic control, endocrine factors, adipose tissue distribution), psychosocial factors, and differences in diabetes care, may contribute to the observed disparities [58]. The observed higher excess risk for AMI in women with diabetes compared to men with diabetes indicates a necessity for the development of sex-specific screenings, prevention programs, and therapeutic concepts.

### Time trends

The majority of the included studies found a reduction in the incidence of AMI over time in both men and women without diabetes. However, results concerning the time trends of incidence in the population with diabetes were inconsistent. The reduction in the incidence of AMI may be attributed to improved management of risk factors for myocardial infarction [59]. Effective and contemporary blood pressure and dyslipidemia therapy in combination with improved lifestyle factors, such as weight reduction, increased physical activity, dietary changes, stress management, and smoking cessation, were described as important factors for the prevention of AMI in people with diabetes [59–63]. Nevertheless, personalized glucose-lowering therapy coupled with adequate blood glucose control remains paramount for individuals with diabetes [62, 64]. Recent clinical trials and population-based studies found that new classes of glucose-lowering drugs such as sodium glucose transport protein 2 inhibitors (SGLT2i) and glucagon-like peptide-1 receptor agonists (GLP-1 RA) may reduce CVD risk in patients with type 2 diabetes [65–68]. The American Diabetes Association (ADA) has recommended these drug classes as first-line therapy for persons with type 2 diabetes and CVD or renal comorbidity since 2022 [62]. However, the effect of these new classes of glucose-lowering drugs on the time trend of AMI was not discussed in detail in the studies reviewed. We assume that the unchanged or increased time trends of incidence in people with diabetes may be partially attributable to the improved diagnostics of AMI using modern ECG and angiography techniques, in combination with the use of more sensitive bio-markers such as troponin since the late 1990 s and early 2000s. In particular, people with diabetes due to cardiac autonomic neuropathy are affected more often by silent AMI with atypical symptoms and little or no pain than people without diabetes and benefit considerably from methods providing improved AMI diagnosis over time.

The findings regarding time trends of estimates comparing people with and without diabetes in the reviewed studies were contradictory. The results showed that most countries have failed to achieve the overarching goal of the 1989 St. Vincent Declaration of reducing the gap between people with and without diabetes. Therefore, greater efforts to prevent AMI and its risk factors among people with diabetes are needed, including integrating cardiovascular risk surveillance into diabetes care and implementing tailored prevention strategies [69, 70]. However, unchanged estimates comparing people with and without diabetes should not necessarily be interpreted in a negative manner. Such findings may also reflect parallel improvements between people with and without diabetes [4, 71, 72].

### Strengths and limitations

A key strength of our review is the systematic search approach with clearly determined search strategies. We only included studies reporting estimates within the population at risk, i.e., among people with diabetes. This approach was adapted independently of the change in diabetes prevalence and thus allowed a more correct interpretation of the results. Furthermore, our systematic review was based on population-based studies and therefore had higher generalizability than RCTs or clinic-based studies. Moreover, we reported results that took the different types of diabetes into account. Finally, we conducted the review without time restrictions, thus providing an overview of the incidence and risk of AMI among people with and without diabetes, and corresponding time trends over the past 40 years.

However, some limitations should be mentioned. Firstly, the majority of studies provided data on people with type 2 diabetes or on study populations where the type of diabetes was not specified. Due to the small number of studies investigating type 1 diabetes, we were unable to accurately analyze differences between people with type 1 and type 2 diabetes. Given that up to 90% of the population with diabetes is comprised of people with type 2 diabetes, our findings are predominantly applicable to such individuals. Secondly, some discrepancies in the estimates comparing the incidence of AMI among people with and without diabetes were attributable to the use of different estimators (RR or HR). However, all estimates permit valid statements regarding the disparity between the population with and without diabetes. Moreover, the time trend of the ratio is not affected by the estimation method. Thirdly, not all studies reviewed were able to adjust results for important lifestyle factors such as smoking, hypertension, and lipid profiles. Nevertheless, we have provided information on adjustment factors in the relevant tables. Fourthly, we only included studies published in the English language and therefore cannot exclude the possibility of publication (language) bias. It is thus possible that relevant non-English language studies, e.g., from Asia or Latin America were not considered. Fifthly, given the unfeasibility of conducting a reliable meta-analysis, the evaluation of potential publication bias by means of formal statistical tests was rendered inadequate. Consequently, the possibility of publication bias cannot be entirely discounted. Finally, the majority of the studies included in this review originate from high-income countries in Europe and the USA. Consequently, our findings are only representative of these regions and do not reflect a global perspective.

## Conclusions

The findings of this systematic review indicate a consistently higher incidence of AMI in people with diabetes compared to those without diabetes. The majority of studies reported a higher incidence in men than women, irrespective of diabetes status. However, women exhibited an unfavorable relative risk when people with and without diabetes were compared. Almost all studies reported decreasing time trends in people without diabetes, although time trends regarding the incidence of AMI in people with diabetes were inconsistent. Thus, the time trends of estimates comparing people with and without diabetes were also different. These findings highlight an ongoing disparity in cardiovascular outcomes and underscore the necessity for additional initiatives to prevent coronary heart disease in people with diabetes. Wide variation in study results may be at least partly explained by the different methodological approaches applied by the included studies and ought to be considered when interpreting findings. We recommend that future studies reporting the incidence of AMI employ a comparable study design, particularly with regard to the definition of AMI and the population with diabetes. It would be beneficial for future studies to include data from countries outside of Europe and the USA.

## Supplementary Information

Additional file 1: Supplementary Table S1. PRISMA 2020 Checklist.

Additional file 2: Search Strategies.

Additional file 3: Supplementary Table S2. Eligibility criteria.

Additional file 4: Supplementary Table S3. Risk of bias assessment across the included studies.

Additional file 5: Supplementary Figure S1. Estimates comparing women with and without diabetes.

Additional file 6: Supplementary Figure S2. Estimates comparing men with and without diabetes.

## Data Availability

The authors confirm that the data supporting the findings of this study are available within the article.
