# Supplementary material for: Incidence of acute myocardial infarction in people with diabetes compared to those without diabetes: a systematic review
Source: Syst Rev. 2026 Feb 9;15:90. doi: 10.1186/s13643-026-03089-x (PMC13011307; doi:10.1186/s13643-026-03089-x)
Supplement: Supplementary file 2 — Additional file 2: Search Strategies. [file 13643_2026_3089_MOESM2_ESM.docx]

**Supplemental Material**

**Search strategies**

**Project: Incidence of myocardial infarction in the population with and without diabetes: a systematic review**

**Medline (Ovid SP)**

**Population/risk factor**

1. Diabetes Mellitus/
2. Diabetes Mellitus, Type 1/
3. Diabetes Mellitus, Type 2/
4. diabet*.tw.
5. (IDDM or NIDDM or T1D? or T2D?).tw.
6. or/1-5

**Complications/Outcomes (MI)**

1. exp Myocardial Infarction/
2. (heart infarct* or heart attack?).tw.
3. myocardial infarct*.tw.
4. ((cardiovascular or coronary) adj (disease or event? or accident?)).tw.
5. (coronary artery disease? or coronary heart disease?).tw.
6. or/7-11

**Study designs/Epidemiology**

1. exp Myocardial Infarction/ep,et
2. Coronary Disease/ep,et
3. epidemiolog*.tw.
4. (inciden* adj3 (heart infarct* or heart attack? or myocardial infarct*)).tw.
5. (inciden* adj3 ((cardiovascular or coronary) adj (disease or event? or accident?))).tw.
6. ((follow or followed) adj6 (population or cohort? or study or year? or prospectiv* or retrospectiv* or long*)).tw.
7. ((population or community) adj based).tw.
8. ((prospectiv* or retrospectiv* or historical) adj3 stud*).tw.
9. diabetic population?.tw.
10. cohort?.tw.
11. (survey? adj6 (health or national or population or community)).tw.
12. ((nation* or administrat* or worldwide or multinational or state or states or region* or geograph*) adj8 (database or health or survey or statistic* or record? or study or claims or sample or survey? or data or register? or registr*)).tw.
13. or/13-24

**Measures of outcomes/analyses**

1. Incidence/
2. exp Survival Analysis/
3. incidence?.tw.
4. (relative risk? or risk ratio? or RR or RRs or hazard ratio? or HR or HRs or odds ratio? or ORs).tw.
5. ((rate or rates) adj4 (myocard* or infarct*)).tw.
6. or/26-30
7. 6 and 12 and 25 and 31

**Exlusion of trials, genetics, animals, interventions…**

1. (randomized controlled trial or controlled clinical trial or comment or letter or editorial or case reports).pt.
2. 32 not 33
3. exp animals/ not humans/
4. 34 not 35
5. (trial or randomi*).ti.
6. 36 not 37
7. ((coronary or cardiac or hand or obesity or cerebrovascular or gynecologic or bypass or heart or bariatric or vascular or neurological or cancer or orthopaedic or thoracic or revasculari?ation or spine or vitreoretinal) adj surgery).ti.
8. 38 not 39
9. (genetic* or gene? or postoperativ* or hiv or hepatitis or pulmonary or bladder or urinary or catheter* or acromegaly or skin or dermatitis or sleep* or erectile or guideline? or molecule? or arthritis or valve? or atrial or arterectomy or endarterectomy or colonoscopy or parkinson* or carotid or fish or prostat* or radiation or hyperthyroid* or periodont* or chocolate or transplant* or pregnanc* or pregnant or psoriasis or epilep* or inflammat* or gout or pneumoni* or fatty liver or ejection fraction or biomarker* or anxiety or bleeding or helicobacter or cell? or osteoarthritis or statin? or warfarin or sulfonyl* or sulphonyl* or dipeptidyl or SGLT* or glucagon or pioglitazone or vildagliptin or aspirin or sodium glucose or dpp-4 or beta block* or antibod* or sitagliptin or dapagliflozin or cocaine or androgen or asthma* or polymorphi* or bromocriptine or tinnitus or imaging or immune or visual or meat or endoscop* or vitamin or cortisol or hysterectom* or fungal or acid or hearing or necrosis or homocystein* or cytokine* or optic* or prasugrel or clopidogrel or infectio* or adenoma? or ezetimibe or dairy or radiotherapy or retinal vein or glomerular or polymer or polycystic or iodine or steroid or eluting or systolic or proton or ventricular or perioperativ* or thyroid or inhibitor? or vaccine? or maternal or peptide? or suicid* or galectin or restless or antihypertens* or fibrin* or hormon? or tissue or claudication or cataract or lung or emergenc* or ambulance or subtype* or troponin or aortic or extracell* or grafting or nephrolithiasis or craniopharyng* or intelligence or atherectomy or corneal or tooth or teeth or septic* or syncop* or oxygen or glaucoma or clipping or mitochondrial or rodent? or mice or rats or antipsyc* or pituitary or vasculitis or gravis or gastrectom* or defibrillat* or neck or testosterone or headache or hypnoti* or dabigatran or Alzheimer* or earthquake or antenatal or skelet* or colectom* or pollution or sweet* or fatty acid? or miscarr* or retinoblast* or ankylosing or thiazoli* or amino* or pitavastatin or mammary or growth factor or brachial or arteriopath* or personality or fistula or tracheal or leptin or adipocytokin* or thrombolysis or hyponatrem* or virus or viral or influenza or migraine or COPD or esophag* or cholangitis or irritable or tuberculos* or infertil* or leucocyt* or herpes or genotype? or cliostalzol or poisoning or mannitol or lupus or orlistat or delirium or calcium or white matter or wound? or incontinen* or antithrombo* or valsartan or doppler or apolipoprotein or hyperparathyroid* or heart rate or injection? or sarcopen* or schizophren* or saturated fat? or sepsis or dental or aliskiren or vertigo or an?emia or hand grip or graft or aorta or hypothyroid* or prescription? or nocturia or palsy or oedema or edema or contracept* or ramipril or estrogen or transcatheter or fat intake or leuko* or exenatide or arthroplast* or dengue or moyamoya or rotator or neoplas* or pancreatitis or ticagrelor or fertility or deep vein or prosomastatin or nasal or PubMed or magnesium or glioblastom* or pacemaker? or saxagliptin or ventilation or tracheostom*or balloon or knee or leukemia or shoulder or staphylococcus or cancer survivor* or grip strenght or antioxidan* or vision loss or amnesia or algorithm? or surgical complication? or bariatric or grafts or lymphocyt* or cocoa or oophorectom* or laparosco* or yoga or antiretroviral or enteral or gastritis or gylburide or fever or endocarditis or aflibercept or fibromyalgia or hydrocephalus or cervical or atorvastatin or ranibizumab or myopia or hip replace* or hip fracture? or alopecia or breast cancer or allopurinol or splenectomy or retinal vessel? or television or nifedipine or readmission? or fenofibrate or polypharma* or rheumat* or potassium or interleukin or urokinase or disturb* or cognitive test or atrophy or spinal cord or polyphenol* or zolpidem or mastectom* or enzym* or cilostazol or ovarian or colorectal or abuse or bowel or colesevelam or mutation? or duloxetine or transaminase or methotrexate or shock or height or stiffness or discectomy or scoliosis or rosiglitazone or case series or duloxetine or neurosurgery or diarrhoea or cyst? or chagas or sibling or pyrexia or dysphagia or ABCD2 or hydroxy* or psychiatri* or transfusion? or metalloproteinase? or h?ematuria or admission? or percutaneous or bipolar disorder? or prosomatostatin or nonadherence or oral or h?emodialysis or willebrand or birth weight or proteinuria or phospholipase or osteoglycin or opium or pemphigoid or multiple sclerosis or heavy drink* or glucitol or perfusion or angioplasty or manganese or estradiol or bilirubin or earlobe? or coffee or CABG or vertebral fracture? or hyperuricemi* or hypotherm* or statement or highway? or revasculari?ation? or dizziness or butter or laryngotrach* or tracheostom* or intravenous or neurotroph* or novel marker? or acute kidney injur* or an?esthe* or interarm or multivitamin* or liraglutide or lixisenatide or chili or treatment failure? or gallbladder or elbow? or gastrointestin* or recanali?atio* or down syndrome or empagliflozin or case stud* or radius fracture? or tv viewing or glyburide or peritoneal dialysis or glibenclamide or prenatal or semen or poststroke or kidney recipient? or contamination or menarche or kidney biopsy or lymphoma or recommendation? or pancreatic cancer or pesticide? or preeclampsia or renal failure or terrorist? or organic food or endotracheal or cooking or anorexia or celiac or febuxostat or aldosteronism or toothbrush* or glomerulonephr* or kidney stone? or vasospastic angina or methylation or hyperfiltration or sudden cardiac death? or diagnostic error? or immunosupress* or ramadan or sexual violence or zeaxanthin or arsenic or herbal or psoria* or osteomyelitis or hidradenitis or deafness or panic disorder? or bisphenol or byphenyl? or rosacea or linagliptin or fried food? or steatohepatitis or weather or resveratrol or h?emochromatosis or phone based intervention? or severe mental illness* or language barriers or nurse facilitated intervention or biliary cirrhosis or polyneuropath* or venous thromboembolism? or hashimoto thyroid* or retinal artery occlusion or insulin secretagogues or potato or rhinitis or vertrebral artery or myocardial deformation? or interdental or visfatin or stillbirth or peglispro or whole grain? or reperfusion therapy or digoxin or preterm birth? or diverticul* or insomnia or olmesartan or NSAID? or p wave or t wave or pressure ulcer? or renal replacement therap* or nephrotoxicity or gallstones or methionine or stoma or bioresorbable or rehospitali? or tuberous sclerosis or breast reconstruction? or retinal vascular caliber or retinal arteriolar caliber or treatment resistant hypertension or constipation or thermoregulat* or cilexetil or autopsy or betel nut? or snoring or pravastatin or mammography or binge drinking or fine particles or trials or bone mineral density or sirolimus or paclitaxel or stents or erythrocyte* or breast feeding or cardiact stress testing or eplerenone or inhaled insulin or treadmill testing or shift work* or stent or ivabradine or metoprolol or sarcoidosis or incretin or dust or amalgam or arterial injur* or surgical mortality).ti.
10. 40 not 41

**Embase**

**Population/Risk Factor**

1. diabetes mellitus/
2. insulin dependent diabetes mellitus/
3. non insulin dependent diabetes mellitus/
4. diabet*.tw.
5. (IDDM or NIDDM or T1D? or T2D?).tw.
6. or/1-5

**Complications/Outcomes (MI)**

1. heart infarction/
2. (heart infarct* or heart attack?).tw.
3. myocardial infarct*.tw.
4. ((cardiovascular or coronary) adj (disease or event? or accident?)).tw.
5. (coronary artery disease? or coronary heart disease?).tw.
6. or/7-11

**Studydesigns/Epidemiology**

1. heart infarction/ep
2. coronary artery disease/ep
3. epidemiolog*.tw.
4. (inciden* adj3 (heart infarct* or heart attack? or myocardial infarct*)).tw.
5. (inciden* adj3 ((cardiovascular or coronary) adj (disease or event? or accident?))).tw.
6. ((follow or followed) adj6 (population or cohort? or study or year? or prospectiv* or retrospectiv* or long*)).tw.
7. ((population or community) adj based).tw.
8. ((prospectiv* or retrospectiv* or historical) adj3 stud*).tw.
9. diabetic population?.tw.
10. cohort?.tw.
11. (survey? adj6 (health or national or population or community)).tw.
12. ((nation* or administrat* or worldwide or multinational or state or states or region* or geograph*) adj8 (database or health or survey or statistic* or record? or study or claims or sample or survey? or data or register? or registr*)).tw.
13. or/13-24

**Measures of outcomes/analyses**

1. incidence/
2. survival analysis/
3. incidence?.tw.
4. (relative risk? or risk ratio? or RR or RRs or hazard ratio? or HR or HRs or odds ratio? or ORs).tw.
5. ((rate or rates) adj4 (myocard* or infarct*)).tw.
6. or/26-30
7. 6 and 12 and 25 and 31
8. limit 32 to embase

**Exlusion of trials, genetics, animals, interventions…**

1. randomized controlled trial/ or double blind procedure/ or case report/
2. 33 not 34
3. (trial? or randomi*).ti.
4. 35 not 36
5. (comment or letter or editorial or conference).pt.
6. 37 not 38
7. ((coronary or cardiac or hand or obesity or cerebrovascular or gynecologic or bypass or heart or bariatric or vascular or neurological or cancer or orthopaedic or thoracic or revasculari?ation or spine or vitreoretinal) adj surgery).ti.
8. 39 not 40
9. (genetic* or gene? or postoperativ* or hiv or hepatitis or pulmonary or bladder or urinary or catheter* or acromegaly or skin or dermatitis or sleep* or erectile or guideline? or molecule? or arthritis or valve? or atrial or arterectomy or endarterectomy or colonoscopy or parkinson* or carotid or fish or prostat* or radiation or hyperthyroid* or periodont* or chocolate or transplant* or pregnanc* or pregnant or psoriasis or epilep* or inflammat* or gout or pneumoni* or fatty liver or ejection fraction or biomarker* or anxiety or bleeding or helicobacter or cell? or osteoarthritis or statin? or warfarin or sulfonyl* or sulphonyl* or dipeptidyl or SGLT* or glucagon or pioglitazone or vildagliptin or aspirin or sodium glucose or dpp-4 or beta block* or antibod* or sitagliptin or dapagliflozin or cocaine or androgen or asthma* or polymorphi* or bromocriptine or tinnitus or imaging or immune or visual or meat or endoscop* or vitamin or cortisol or hysterectom* or fungal or acid or hearing or necrosis or homocystein* or cytokine* or optic* or prasugrel or clopidogrel or infectio* or adenoma? or ezetimibe or dairy or radiotherapy or retinal vein or glomerular or polymer or polycystic or iodine or steroid or eluting or systolic or proton or ventricular or perioperativ* or thyroid or inhibitor? or vaccine? or maternal or peptide? or suicid* or galectin or restless or antihypertens* or fibrin* or hormon? or tissue or claudication or cataract or lung or emergenc* or ambulance or subtype* or troponin or aortic or extracell* or grafting or nephrolithiasis or craniopharyng* or intelligence or atherectomy or corneal or tooth or teeth or septic* or syncop* or oxygen or glaucoma or clipping or mitochondrial or rodent? or mice or rats or antipsyc* or pituitary or vasculitis or gravis or gastrectom* or defibrillat* or neck or testosterone or headache or hypnoti* or dabigatran or Alzheimer* or earthquake or antenatal or skelet* or colectom* or pollution or sweet* or fatty acid? or miscarr* or retinoblast* or ankylosing or thiazoli* or amino* or pitavastatin or mammary or growth factor or brachial or arteriopath* or personality or fistula or tracheal or leptin or adipocytokin* or thrombolysis or hyponatrem* or virus or viral or influenza or migraine or COPD or esophag* or cholangitis or irritable or tuberculos* or infertil* or leucocyt* or herpes or genotype? or cliostalzol or poisoning or mannitol or lupus or orlistat or delirium or calcium or white matter or wound? or incontinen* or antithrombo* or valsartan or doppler or apolipoprotein or hyperparathyroid* or heart rate or injection? or sarcopen* or schizophren* or saturated fat? or sepsis or dental or aliskiren or vertigo or an?emia or hand grip or graft or aorta or hypothyroid* or prescription? or nocturia or palsy or oedema or edema or contracept* or ramipril or estrogen or transcatheter or fat intake or leuko* or exenatide or arthroplast* or dengue or moyamoya or rotator or neoplas* or pancreatitis or ticagrelor or fertility or deep vein or prosomastatin or nasal or PubMed or magnesium or glioblastom* or pacemaker? or saxagliptin or ventilation or tracheostom*or balloon or knee or leukemia or shoulder or staphylococcus or cancer survivor* or grip strenght or antioxidan* or vision loss or amnesia or algorithm? or surgical complication? or bariatric or grafts or lymphocyt* or cocoa or oophorectom* or laparosco* or yoga or antiretroviral or enteral or gastritis or gylburide or fever or endocarditis or aflibercept or fibromyalgia or hydrocephalus or cervical or atorvastatin or ranibizumab or myopia or hip replace* or hip fracture? or alopecia or breast cancer or allopurinol or splenectomy or retinal vessel? or television or nifedipine or readmission? or fenofibrate or polypharma* or rheumat* or potassium or interleukin or urokinase or disturb* or cognitive test or atrophy or spinal cord or polyphenol* or zolpidem or mastectom* or enzym* or cilostazol or ovarian or colorectal or abuse or bowel or colesevelam or mutation? or duloxetine or transaminase or methotrexate or shock or height or stiffness or discectomy or scoliosis or rosiglitazone or case series or duloxetine or neurosurgery or diarrhoea or cyst? or chagas or sibling or pyrexia or dysphagia or ABCD2 or hydroxy* or psychiatri* or transfusion? or metalloproteinase? or h?ematuria or admission? or percutaneous or bipolar disorder? or prosomatostatin or nonadherence or oral or h?emodialysis or willebrand or birth weight or proteinuria or phospholipase or osteoglycin or opium or pemphigoid or multiple sclerosis or heavy drink* or glucitol or perfusion or angioplasty or manganese or estradiol or bilirubin or earlobe? or coffee or CABG or vertebral fracture? or hyperuricemi* or hypotherm* or statement or highway? or revasculari?ation? or dizziness or butter or laryngotrach* or tracheostom* or intravenous or neurotroph* or novel marker? or acute kidney injur* or an?esthe* or interarm or multivitamin* or liraglutide or lixisenatide or chili or treatment failure? or gallbladder or elbow? or gastrointestin* or recanali?atio* or down syndrome or empagliflozin or case stud* or radius fracture? or tv viewing or glyburide or peritoneal dialysis or glibenclamide or prenatal or semen or poststroke or kidney recipient? or contamination or menarche or kidney biopsy or lymphoma or recommendation? or pancreatic cancer or pesticide? or preeclampsia or renal failure or terrorist? or organic food or endotracheal or cooking or anorexia or celiac or febuxostat or aldosteronism or toothbrush* or glomerulonephr* or kidney stone? or vasospastic angina or methylation or hyperfiltration or sudden cardiac death? or diagnostic error? or immunosupress* or ramadan or sexual violence or zeaxanthin or arsenic or herbal or psoria* or osteomyelitis or hidradenitis or deafness or panic disorder? or bisphenol or byphenyl? or rosacea or linagliptin or fried food? or steatohepatitis or weather or resveratrol or h?emochromatosis or phone based intervention? or severe mental illness* or language barriers or nurse facilitated intervention or biliary cirrhosis or polyneuropath* or venous thromboembolism? or hashimoto thyroid* or retinal artery occlusion or insulin secretagogues or potato or rhinitis or vertrebral artery or myocardial deformation? or interdental or visfatin or stillbirth or peglispro or whole grain? or reperfusion therapy or digoxin or preterm birth? or diverticul* or insomnia or olmesartan or NSAID? or p wave or t wave or pressure ulcer? or renal replacement therap* or nephrotoxicity or gallstones or methionine or stoma or bioresorbable or rehospitali? or tuberous sclerosis or breast reconstruction? or retinal vascular caliber or retinal arteriolar caliber or treatment resistant hypertension or constipation or thermoregulat* or cilexetil or autopsy or betel nut? or snoring or pravastatin or mammography or binge drinking or fine particles or trials or bone mineral density or sirolimus or paclitaxel or stents or erythrocyte* or breast feeding or cardiact stress testing or eplerenone or inhaled insulin or treadmill testing or shift work* or stent or ivabradine or metoprolol or sarcoidosis or incretin or dust or amalgam or arterial injur* or surgical mortality).ti.
10. 41 not 42

**LILACS (iAHX interface)**

diabet$ AND inciden$ AND (((infart$ OR enfarto$) AND miocardio) OR ((acidente$ OR accidente$ OR evento$) AND (vascular$ OR cardiovascular$)
