# Supplementary material for: Incidence of acute myocardial infarction in people with diabetes compared to those without diabetes: a systematic review
Source: Syst Rev. 2026 Feb 9;15:90. doi: 10.1186/s13643-026-03089-x (PMC13011307; doi:10.1186/s13643-026-03089-x)
Supplement: Supplementary file 3 — Additional file 3: Supplementary Table S2. Eligibility criteria. [file 13643_2026_3089_MOESM3_ESM.docx]

Supplementary Table 2 **Eligibility criteria**

| **Inclusion criteria** | **Exclusion criteria** |
| --- | --- |
| **Types of studies** | |
| Population-based cohort studies | Non population-based studies (e.g., hospital-centered studies, RCT)  Cross-sectional studies |
| **Study population** | |
| People with diabetes compared to people without diabetes | Only people with diabetes  General population without stratification by diabetes status |
| **Outcome AMI** | |
| Both fatal and non-fatal AMI | Only non-fatal AMI  Only fatal AMI |
| **Epidemiological measures** | |
| Incidence rate  Cumulative incidence  Relative risk  Hazard ratio | Prevalence  Prevalence ratio  Odds ratio |
| **Statistical methods** | |
| Age-(sex)-adjusted incidence rate | Crude incidence rate |
| Incidence rates should be reported in relation to the population at risk (people with diabetes) | Incidence rates were reported in relation to the total population (people with and without diabetes) |
| **Language** | |
| English language | Language other than English |
