# Supplementary material for: Incidence of acute myocardial infarction in people with diabetes compared to those without diabetes: a systematic review
Source: Syst Rev. 2026 Feb 9;15:90. doi: 10.1186/s13643-026-03089-x (PMC13011307; doi:10.1186/s13643-026-03089-x)
Supplement: Supplementary file 4 — Additional file 4: Supplementary Table S3. Risk of bias assessment across the included studies. [file 13643_2026_3089_MOESM4_ESM.docx]

Supplementary Table 3 Risk of bias assessment across the included studies

| Study | D1 | D2 | D3 | D4 | D5 | D6 |
| --- | --- | --- | --- | --- | --- | --- |
| Ballotari, P, 2017, Italy |  |  |  |  |  |  |
| Barengo, N, 2008, Finland |  |  |  |  |  |  |
| Carson, A, 2014, U.S. |  |  |  |  |  |  |
| de Jong, M, 2020, UK |  |  |  |  |  |  |
| Dybjer, E, 2021, Sweden |  |  |  |  |  |  |
| Folsom, A, 1997, U.S. |  |  |  |  |  |  |
| Fujishima, M, 1996, Japan |  |  |  |  |  |  |
| Haffner, S, 1998, Finland |  |  |  |  |  |  |
| Hyvarinen, M, 2009, Finland |  |  |  |  |  |  |
| Icks, A, 2009, Germany |  |  |  |  |  |  |
| Juutilainen, A, 2004, Finland |  |  |  |  |  |  |
| Liu, F, 2017, China |  |  |  |  |  |  |
| Lundberg, V, 1997, Sweden |  |  |  |  |  |  |
| Matuleviciene, V, 2017, Sweden |  |  |  |  |  |  |
| Millett, E, 2018, UK |  |  |  |  |  |  |
| Moe, B, 2015, Norwegen |  |  |  |  |  |  |
| Nørgaard, C, 2022, Denmark |  |  |  |  |  |  |
| Oğuz, A, 2023, Türkiye |  |  |  |  |  |  |
| Rautio, A, 2005, Sweden |  |  |  |  |  |  |
| Read, SH, 2019, UK |  |  |  |  |  |  |
| Rosengren, A, 1989, Sweden |  |  |  |  |  |  |
| Saeed, M, 2022, Norway |  |  |  |  |  |  |
| Saito, I, 2011, Japan |  |  |  |  |  |  |
| Schramm, T, 2008, Denmark |  |  |  |  |  |  |
| Tancredi, M, 2019, Sweden |  |  |  |  |  |  |
| Vimalananda, V, 2014, U.S. |  |  |  |  |  |  |
| Wannamethee, S, 2011, UK |  |  |  |  |  |  |
| Wright, A, 2019, UK |  |  |  |  |  |  |

Low risk of bias

High risk of bias

Unclear risk of bias*

Time trend was not analyzed

Domains:

D1 Study population

D2 Definition of diabetes

D3 Definition of AMI outcome

D4 Absolute number of AMI cases

D5 Precision and consistency of results

D6 Reporting of the time trend

* The risk of bias was assessed as unclear if the available information was insufficient to accurately determine the level of risk of bias.

Assessment of risk of bias

| **Assessment items** | **Low risk of bias** | **High risk of bias** |
| --- | --- | --- |
| Study population | Nationwide study | Regional/local study |
| Definition of diabetes | Medical diagnosis, blood glucose or HbA1c measurement, | Self-reported diagnosis |
| Definition of AMI outcome | Clear definition of AMI | Absence of a clear definition of AMI |
| Absolute number of AMI cases | Sufficient number > 10 cases | Very small number ≤ 10 cases |
| Precision and consistency of results | 95% CI was narrow | 95% CI was wide or 95% CI was not reported |
| Time trend | Time trend was reported using multivariate regression models | Time trend was reported only descriptive without appropriate statistical models |
