# Supplementary figures and images for: Incidence of acute myocardial infarction in people with diabetes compared to those without diabetes: a systematic review
Source: Syst Rev. 2026 Feb 9;15:90. doi: 10.1186/s13643-026-03089-x (PMC13011307; doi:10.1186/s13643-026-03089-x)

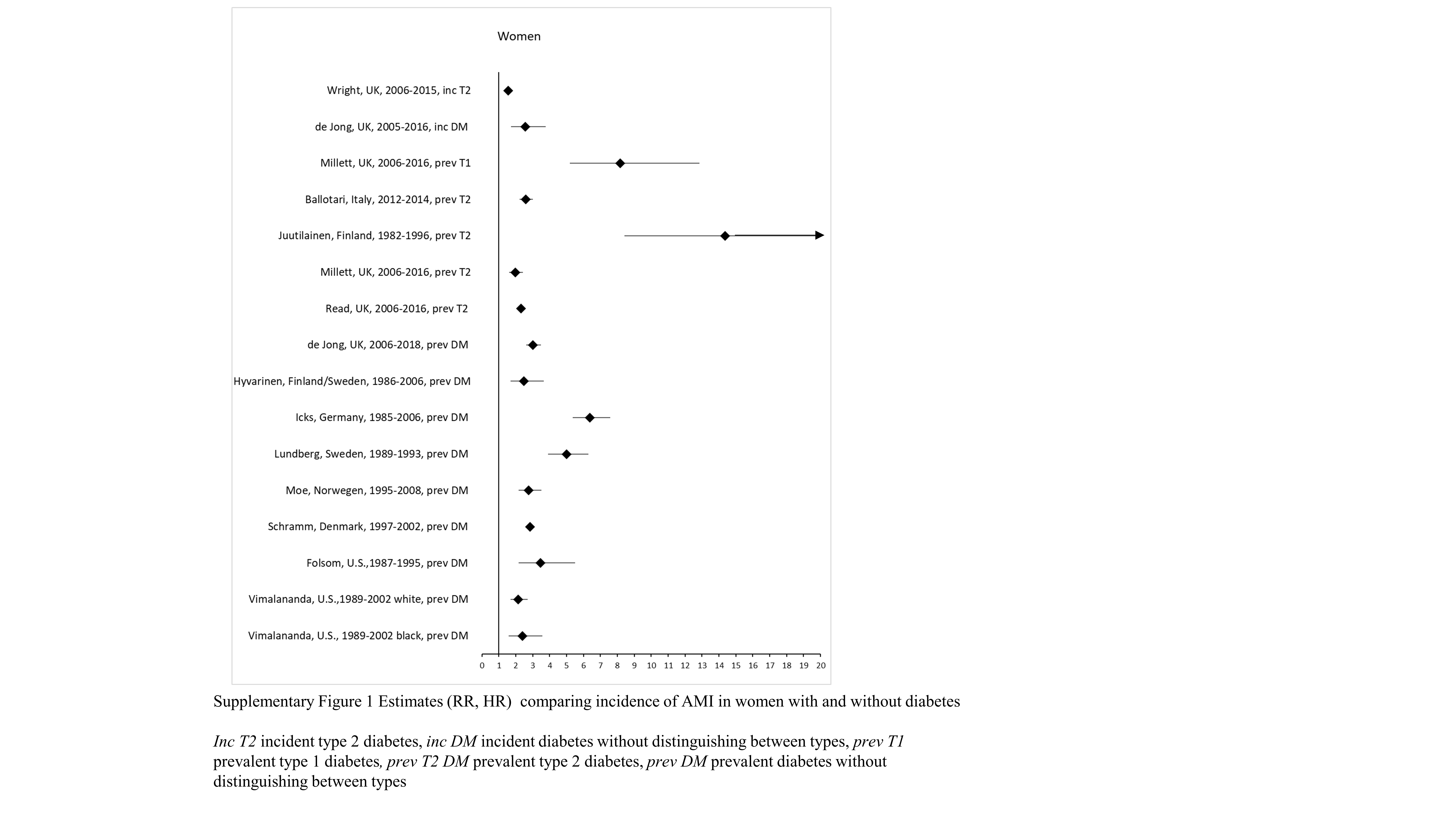

Supplement: Supplementary file 5 — Additional file 5: Supplementary Figure S1. Estimates comparing women with and without diabetes. [file 13643_2026_3089_MOESM5_ESM.tif]

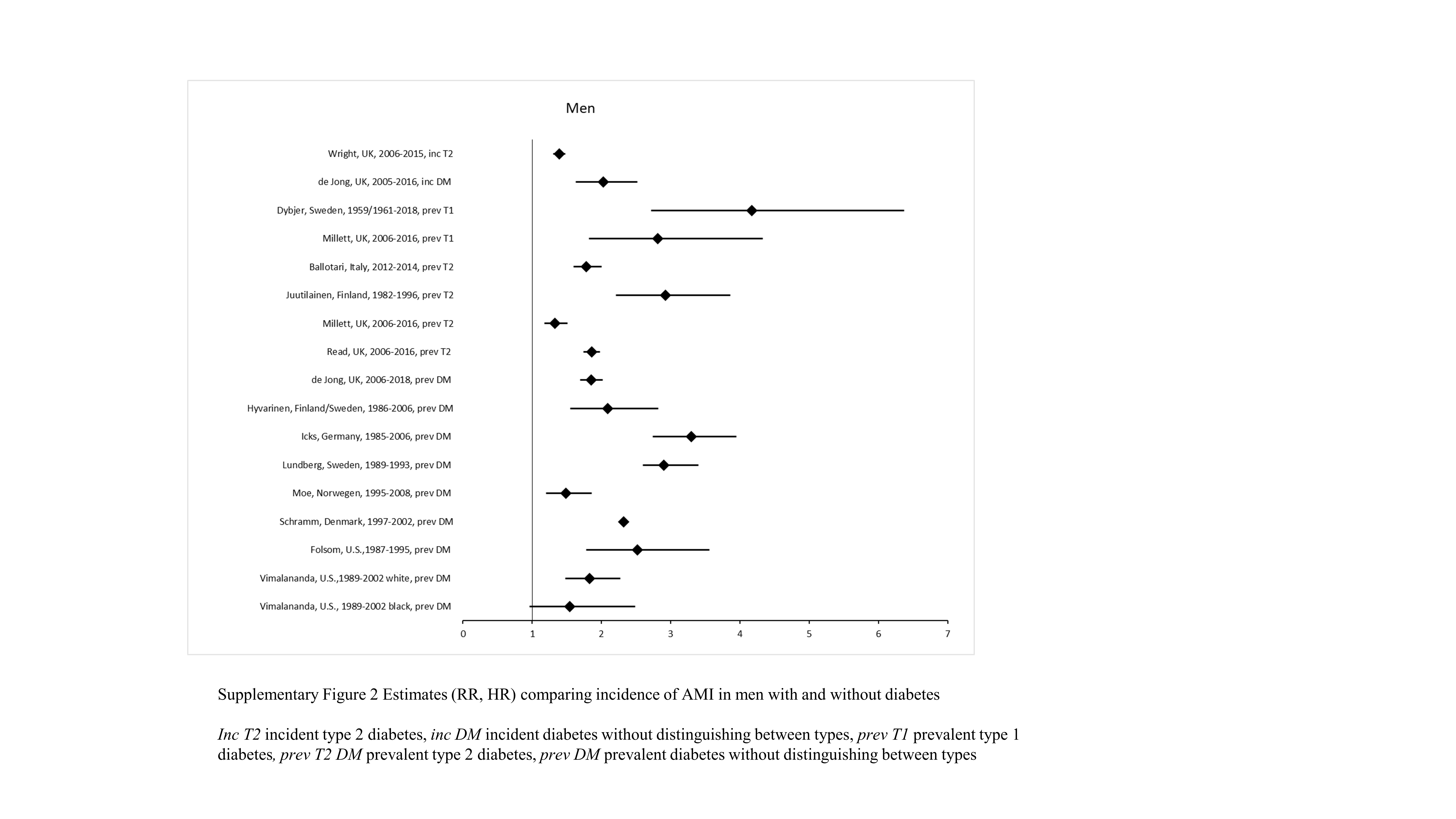

Supplement: Supplementary file 6 — Additional file 6: Supplementary Figure S2. Estimates comparing men with and without diabetes. [file 13643_2026_3089_MOESM6_ESM.tif]
